# Study protocol for a hospital-to-home transitional care intervention for older adults with multiple chronic conditions and depressive symptoms: a pragmatic effectiveness-implementation trial

**DOI:** 10.1186/s12877-020-01638-0

**Published:** 2020-07-10

**Authors:** Maureen Markle-Reid, Carrie McAiney, Rebecca Ganann, Kathryn Fisher, Amiram Gafni, Alain P. Gauthier, Gail Heald-Taylor, Janet McElhaney, Jenny Ploeg, Diana J. Urajnik, Ruta Valaitis, Carly Whitmore

**Affiliations:** 1 Aging, Community and Health Research Unit, School of Nursing, McMaster University, 1200 Main Street West, HSC 3N25B, Hamilton, ON L8S 4K1 Canada; 2 Murray Alzheimer Research & Education Program (MAREP), School of Public Health and Health Systems, University of Waterloo,University of Waterloo Research Institute for Aging, University of Waterloo, 200 University Avenue West, Waterloo, ON N2L 3G1 Canada; 3 Department of Health Research Methods, Evidence, and Impact; and Centre for Health Economics and Policy Analysis, McMaster University, 1200 Main Street West, Hamilton, ON L8S 4K1 Canada; 4 School of Human Kinetics, Laurentian University, 935 Ramsey Lake Rd., Sudbury, ON P3E 2C6 Canada; 5 Patient Research Partner, Selkirk, ON Canada; 6 Medical Sciences Division, Northern Ontario School of Medicine, Health Sciences North Research Institute, 41 Ramsey Lake Road, Sudbury, ON P3E 5J1 Canada; 7 Centre for Rural and Northern Health Research, Laurentian University, 935 Ramsey Lake Rd., Sudbury, ON P3E 2C6 Canada

**Keywords:** Older adults, Multiple chronic conditions, Depressive symptoms, Transitional care, Pragmatic effectiveness-implementation trial, Sustainability, Scale-up

## Abstract

**Background:**

Older adults (> 65 years) with multiple chronic conditions (MCC) and depressive symptoms experience frequent transitions between hospital and home. Care transitions for this population are often poorly coordinated and fragmented, resulting in increased readmission rates, adverse medical events, decreased patient satisfaction and safety, and increased caregiver burden. There is a dearth of evidence on best practices in the provision of transitional care for older adults with MCC and depressive symptoms transitioning from hospital-to-home. This paper presents a protocol for a two-armed, multi-site pragmatic effectiveness-implementation trial of Community Assets Supporting Transitions (CAST), an evidence-informed nurse-led six-month intervention that supports older adults with MCC and depressive symptoms transitioning from hospital-to-home. The Collaborative Intervention Planning Framework is being used to engage patients and other key stakeholders in the implementation and evaluation of the intervention and planning for intervention scale-up to other communities.

**Methods:**

Participants will be considered eligible if they are > 65 years, planned for discharged from hospital to the community in three Ontario locations, self-report at least two chronic conditions, and screen positive for depressive symptoms. A total of 216 eligible and consenting participants will be randomly assigned to the control (usual care) or intervention (CAST) arm. The intervention consists of tailored care delivery comprising in-home visits, telephone follow-up and system navigation support. The primary measure of effectiveness is mental health functioning of the older adult participant. Secondary outcomes include changes in physical functioning, depressive symptoms, anxiety, perceived social support, patient experience, and health and social service use and cost, from baseline to 6- and 12-months. Caregivers will be assessed for caregiver strain, depressive symptoms, anxiety, health-related quality of life, and health and social service use and costs. Descriptive and qualitative data from older adult and caregiver participants, and the nurse interventionists will be used to examine implementation of the intervention, how the intervention is adapted within each study region, and its potential for sustainability and scalability to other jurisdictions.

**Discussion:**

A nurse-led transitional care strategy may provide a feasible and effective means for improving health outcomes and patient/caregiver experience and reduce service use and costs in this vulnerable population.

**Trial registration:**

# NCT03157999.

Registration Date: April 4, 2017.

## Background

Transitioning (moving) between hospital and home after a hospital admission can be a stressful and confusing time for patients and their caregivers. By necessity, the transition from hospital to home involves several professionals within and between disciplines and settings, all sharing the responsibility of care for an individual [1]. This results in numerous challenges to providing comprehensive and coordinated care, particularly for older adults (> 65 years) with multiple (> 2) chronic conditions (MCC) and depressive symptoms who experience frequent hospital-to-home transitions [2]. Care transitions for this population are often poorly coordinated and fragmented, resulting in increased readmission rates, adverse medical events, decreased patient satisfaction and safety, and increased caregiver burden [3–5].

These adverse outcomes have been attributed to factors such as lack of patient knowledge about available community-based services resulting in suboptimal or delayed utilization of these services [6], conflicting plans and instructions from different providers [7–10], medication errors [11, 12], lack of support for self-management of their complex chronic conditions [11, 12], lack of support for family caregivers, lack of communication and connections between older adults and health care providers, and untreated or under-treated depressive symptoms [13–15]. Factors related to social and structural determinants of health can further worsen the challenge of managing hospital to home transitions for older adults with MCC and depressive symptoms [16]. This is especially true for older adults with lower socioeconomic status, ethnocultural minorities, or persons living in rural or remote communities [17]. The consequences of poorly executed transitions are far reaching, leading to unnecessary costs to the health care system, and increased stress and burden on older adults with MCC and depressive symptoms and their caregivers. As the population of older adults increases, the number of persons living with MCC is expected to increase. Older adults with MCC are more likely to experience depressive symptoms than older adults without MCC (18). These data highlight the need for new, integrated healthcare delivery models to improve the quality and experience of hospital to home transitions for this vulnerable population.

Transitional care interventions have been recommended to address adverse outcomes in community-living older adults with complex needs transitioning from hospital to home [18–25]. The aim of transitional care is to ensure the coordination and continuity of health care when older adults move across care settings. Improvements in transitional care will simultaneously lead toward more integrated care, by bringing together services, providers, and organizations from across care settings and sectors; ensuring that services are coordinated and complement one another; and sharing information between providers accurately and promptly [1]. The goal is to develop a seamless unified system to ensure continuity of care for older adults managing MCC and depressive symptoms.

Although these Transitional Care Interventions have been linked to several positive outcomes, including decreasing hospital readmissions, the effectiveness of these interventions for older adults with MCC and depressive symptoms is uncertain. Most trials assessing the impact of transitional care interventions have excluded older adults with MCC, omitted mental health considerations such as depressive symptoms, provide little information on cost and implementation strategies, and used weak study designs [26–29]. Most of these studies have focused on acute care readmission rates as the primary measure of effect for many transitional care interventions, with limited attention to patient-relevant outcomes, such as health-related quality of life. Moreover, little is known about how, and within which contexts, care transition interventions and their components are effective. There is a need for evidence-informed best practices on transitional care for older adults with MCC and depressive symptoms since older adults with depression experience persistent health inequities that often lead to negative outcomes, such as reduced health-related quality of life, and high use of healthcare services [30–34].

### CAST study objectives

Our team designed a new hospital-to-home transitional care intervention to address these gaps in knowledge. The Community Assets Supporting Transitions (CAST) is a 6-month tailored patient-and caregiver-centred intervention delivered by a Care Transition Coordinator (CTC) who is a Registered Nurse (RN). The CAST intervention was designed to improve the quality and experience of hospital-to-home transitions by supporting families and caregivers and fostering collaborations between primary care and other interdisciplinary service providers, both within and outside of the health sector, in delivering home and community services. The study builds upon the results of our feasibility study that demonstrated that a 6-month nurse-led mental health promotion intervention for older home care clients with MCC and depressive symptoms, was feasible to implement and resulted in a statistically significant reduction in depressive symptoms, and a significant improvement in mental health and physical functioning [18]. Statistically significant reductions were observed in the use of hospitalization, ambulance service utilization and emergency room visits [18]. The intervention components (home visits, telephone contacts, care coordination) in the feasibility study are comparable to those featured in effective care transitions interventions [35–37]. The purpose of this paper is to provide a detailed description of the CAST study protocol and discuss any anticipated challenges to implementation and how these will be addressed.

### Research questions

The study addresses three research questions:

1. What is the effect of a new, nurse-led hospital-to-home transitional care intervention compared to usual care on health outcomes and costs for older adults with MCC and depressive symptoms and their caregivers?
2. How is the new care transition intervention adapted and implemented in diverse settings?
3. What is required to sustain and scale up the intervention?

It is hypothesized that, compared to usual care, the intervention will result in improvements in health outcomes for older adults with MCC and depressive symptoms while reducing the utilization of expensive healthcare services (e.g., hospitalization).

### Trial design

The research questions are being addressed using a pragmatic randomized controlled trial (RCT), which applies RCT methodology in actual care settings to better inform decisions about the probable benefits, harms and costs of real-world implementation [38, 39]. The design is further classified as a Type II hybrid effectiveness implementation study, which assigns equal weight to assessing program effectiveness and implementation [40, 41]. Hybrid designs are thought to facilitate the transition from research to practice and result in more rapid uptake of effective interventions [40, 41].

## Methods/design

**Fig. 1:**
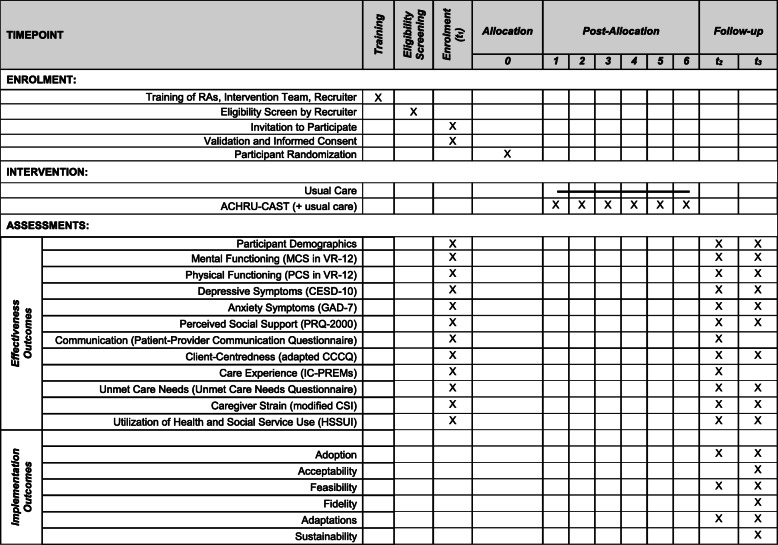
Standard Protocol Items: Recommendations for Interventional Trials (SPIRIT) Checklist: schedule of enrollment, interventions, and assessments)

The CONSORT Standardized Protocol Items: Recommendations for Interventional Trials (SPIRIT) [42] was used to structure the description of the CAST Trial presented in this manuscript and ensure that all relevant information is included. The participant timeline recommended by SPIRIT (42) shows the schedule of enrollment, interventions, and assessments (see Fig. 1) and these are all discussed in greater detail below.

### Study setting

The study is taking place in three distinct communities within the catchment areas of two Regional Health Authorities in Ontario. These three communities were selected because they are diverse with respect to geography (rural/urban), socio-economic, language, and ethno-cultural characteristics. A description of each of the three communities, along with relevant health status indicators, is provided below.

#### Site one

The North East Local Health Integration Network is one of Ontario’s geographically largest LHINs, comprising 44% of Ontario’s land mass and serving over half a million residents [43]. This area of Ontario has the largest Francophone population of any LHIN region and has 41 First Nations and 19 urban and rural aboriginal communities [43]. The North East LHIN has the second highest proportion of older adults (65+) in Ontario (LHIN: 20.4% vs. ON: 16.4%), and the second highest proportion of residents aged 75+ (LHIN: 8.9% vs. ON: 7.2%) [44]. The prevalence rates of North East LHIN residents with a self-reported chronic condition (LHIN: 49.3 per 100 vs. ON: 39.6 per 100) or classified as having multiple chronic conditions (LHIN: 22.2 per 100 vs. ON: 16.2 per 100) are higher than the prevalence rates for Ontario [44]. In 2015–16, a significantly lower proportion of North East LHIN residents reported having very good or excellent self-perceived health compared with Ontarians as a whole (LHIN: 56% vs. ON: 61%); the proportion of North East LHIN residents reporting very good or excellent self-perceived mental health was also significantly lower than that of Ontarians (LHIN: 66% vs. ON: 71%) [44].

#### Site two

The Hamilton Niagara Haldimand Brant (HNHB) LHIN is a population dense region of Ontario with 1.4 million residents [45]. The HNHB LHIN serves more than 260,000 older adults (65+ years) and is projected to increase by 35% by 2025 [46]. The HNHB LHIN has significant socio-economic diversity across the six communities within its catchment area [46]. One of the communities, the second site for the study, reports higher rates of high blood pressure (LHIN: 14.5% vs. ON: 12.9%) and arthritis (LHIN: 20.5% vs. 17.7%) compared to the provincial average [47]. This community reports a lower than provincial average of residents in low-income (LHIN: 7.6% vs. ON: 13.8%), and the highest number of primary care physicians per 10,000 population in comparison with other five communities within the HNHB LHIN (LHIN range: 4.7 to 6.5) [47].

#### Site three

The third site of the study is also within the catchment area of the HNHB LHIN. This community has a greater population than site two and a greater proportion of older adults (24.5%), with 15.7% of those residents living below the low-income threshold [47]. The community also has the second highest rate of older adults living alone and the highest percentage of repeat, unscheduled emergency department visits within 30 days for mental health conditions within the HNHB sub regions (21.2%), similar to the Ontario proportion [47]. This large community, although geographically similar to the second site, reports a lower than average socio-economic status (SES) for residents, and greater population density and ethnocultural diversity [48].

### Eligibility criteria

Study participants will be recruited from one hospital within each of the three sites. Each site aims to recruit 72 patients, yielding a sample size of 216 total participants. Older adult participants will be considered eligible if they meet the following criteria: 1) aged 65 years or older; 2) planned for discharge from hospital to the community (including private residences, retirement homes and transitional care beds); 3) self-report having a diagnosis of at least two chronic conditions; 4) self-report of having experienced depressive symptoms as assessed by a two-item version of the Patient Health Questionnaire (PHQ-2) [49]; 5) resides in one of the study regions and are not planning to move out of the region during the trial; 6) capable of providing informed consent or have a decision-maker who is able to provide informed consent; 7) is competent in English or has an interpreter who is competent in English (this also includes French-speaking individuals or French language interpreters at Site One). The adult caregiver(s) of trial participants, defined as family members or friends who assist with the management of their day-to-day activities by providing emotional, financial or other support, will also be invited to participate in the trial.

The Short Portable Mental Status Questionnaire (SPMSQ) [50] will be used to determine if eligible participants can provide informed consent (inclusion criterion 6). Participants will require a score of 5 or higher on the SPMSQ or have access to a substitute decision-maker. Individuals will be excluded from participation in the study if they are being discharged from hospital to a long-term care home, another hospital or hospital unit, or are not competent to provide informed consent and do not have access to a substitute decision maker.

### Participant flow, assessments and timeline

**Fig. 2:**
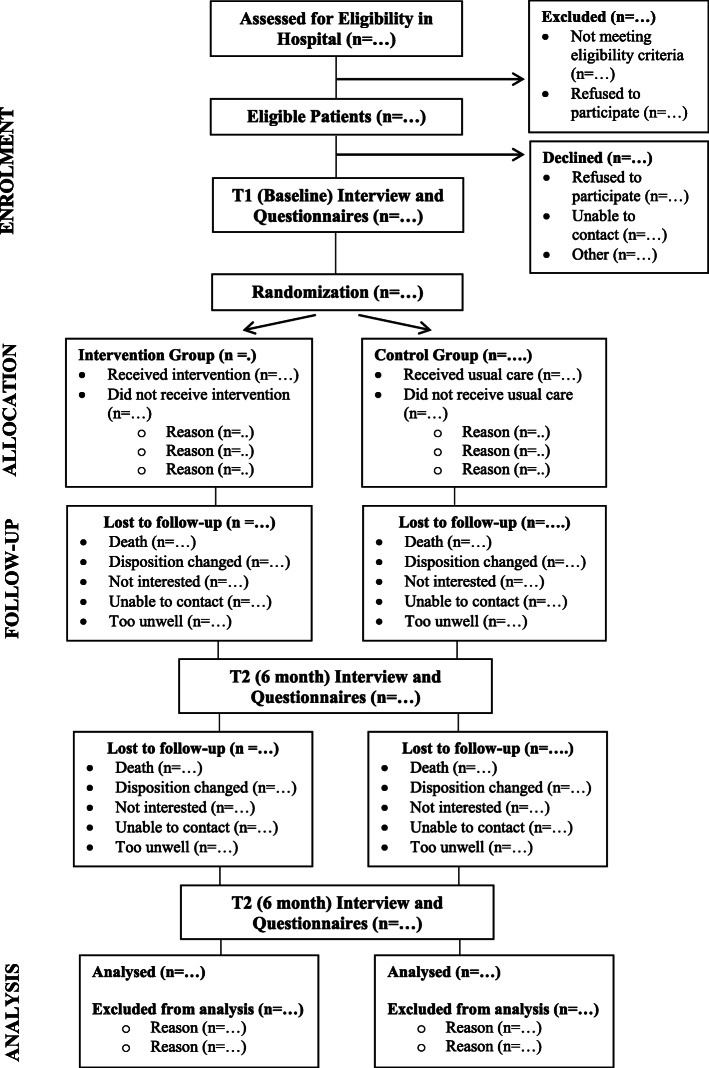
Flow Diagram of Progress through Study Phases

Independent Research Assistants (RAs) will assess participants at baseline within 2 weeks post-hospital discharge (T1) and again at 6 months (T2) and 12 months (T3) after baseline through a structured in-home interview. The study team will aim to complete all assessments at the participant’s place of residence, but completion by telephone is an option if participants prefer. Each assessment will take approximately 1–1 ½ half hours to complete. RAs with previous experience working in community-based settings will be trained in consent and data collection and management procedures. The Research Coordinator will meet with the RAs on a regular basis to address any questions and concerns and ensure consistency of approach across the three sites. The Research Coordinator will check the data at regular intervals during the data collection period in order to keep the number of errors and missing data as low as possible and gather maximum data for analysis. The flow of participants through the study phases will be presented using a flow diagram which conforms to the Consolidated Standards of Reporting Trials (CONSORT) guidelines for pragmatic trial reporting [39] (Fig. 2).

### Sample size

The target sample size of 216 (72 from each of the three sites) was derived from the Mental Component Summary (MCS) score of the VR-12 [51], which assumes a power of 80%, alpha of 0.05, mean MCS score difference of 6.5, standard deviation of 15.0 and 20% attrition, reported in the feasibility study [18].

### Recruitment

Trained recruiters will be identified at participating sites and will receive training about the CAST intervention and eligibility criteria. These individuals will approach potentially eligible patients prior to hospital discharge to determine their interest in learning more about the study. A standardized screening script will be used to introduce the patients to the study. At one site, permission from a member’s circle of care is required before the recruiter approaches the patient.

If the patient agrees to be screened for study eligibility, an eligibility questionnaire will be completed, at which point the recruiter will confirm whether the patient is eligible. Eligible patients will then be asked if they were willing to be contacted about the study following discharge from hospital. Eligible patients who consent to be contacted by the Research Assistant upon discharge from hospital will receive a study information sheet. Participants will be enrolled in the trial after hospital discharge, once written informed consent is provided. Recruitment will occur over a 13-month period across the three study sites.

### Randomization and blinding

Within each study region, participants will be assigned to either the intervention or the usual care group following the collection of baseline data, using stratified permuted block randomization administered by a centralized web-based service (RedCap) to allocate clients at each site to the two groups in accordance with the sequence and using a 1:1 ratio. All study participants will be blinded to their group allocation (usual care or CAST intervention). Research assistants completing further assessments with participants following recruitment will remain blind to group assignments for the duration of data collection. Upon completion of the study at 12-months, participants will receive a mailed debriefing letter describing the two groups and their group allocation.

### Interventions

#### CAST intervention group

The CAST intervention was designed to complement usual care and consists of usual care plus a tailored patient- and caregiver-centred intervention delivered by a Care Transition Coordinator (CTC) who is a Registered Nurse (RN). The CTC will conduct a minimum of two in-home visits along with a minimum of four telephone calls scheduled to accommodate participant needs and preferences over a six-month period.

Throughout the 6 month duration of the intervention, the CTC will: 1) identify the health care professionals involved in the participant’s circle of care and initiate a plan for regular communication and follow-up with these individuals and organizations; 2) identify and manage the patient’s risk factors for depressive symptoms and other chronic conditions in accordance with evidence-based guidelines [52, 53]; 3) provide system navigation support, coordination, and follow up for participants and caregivers; 4) conduct medication review and management in collaboration with participants, primary care providers and pharmacists using evidence-based best practice guidelines [52, 53]; 5) conduct problem-solving therapy with participants and caregivers using Nezu et al.’s [54] manual; 6) implement social and behavioural activation, which involves assisting and encouraging participants to participate in regular physical activity programs tailored to social and behavioural needs; 7) provide patient and caregiver education; and 8) alert the participant’s primary care provider to the presence of depressive symptoms, dementia, delirium, suicidal ideation, or medication issues. These activities will be completed through direct interactions with participants during home visits and phone calls and indirect activities, (e.g., referring participants to health and social services, communicating with healthcare providers in the participant’s circle of care) completed between scheduled appointments.

This pragmatic trial is a tailored intervention with fixed and flexible elements that are adaptable to patient needs and preferences and the local context. Consistent with a pragmatic trial, participants allocated to the CAST intervention, can accept or decline as much of the intervention as they want. Examples of allowable adaptations include removing and/or integrating elements that overlap with routine or usual care. The CTCs will attend an 8-h training session supported by role-appropriate and standardized manuals. Training sessions will be led by the Principal Investigators and the Research Coordinator and include education and role-playing to enhance skills in problem-solving therapy within the context of MCCs.

#### Usual care group

Participants allocated to the control group at discharge from hospital to home will receive usual care. Usual care, which varies from site to site, may include recommendations for the patient to see their primary care provider and/or other medical specialists or access home care and/or other health and social services provided in the community after hospital discharge.

#### Effectiveness measures and data sources

The effectiveness of the CAST intervention on older adults and their caregivers will be evaluated using a range of patient and caregiver-relevant measures. A summary of these measures, including their description, related variables/outcomes, and time point(s) collected, and methods of analysis, are summarized in Table 1.

**Table 1:** CAST Study Outcome Measures Summary

Outcome	Measure	Group*	Timepoint	Method of Analysis
Mental functioning	Mental Component Score of VR-12 (MCS) [51, 55]	PT, CG	T_1_, T_2_, and T_3_	-Descriptive analyses -T1-T2 (Treatment Effect): ANCOVA, complete case and multiple imputation analyses, subgroup analyses, quantile regression -T2-T3 (Sustainability): ANCOVA, complete case and multiple imputation analyses, subgroup analyses
Physical functioning	Physical Component Score (PCS) of VR-12 [51, 55]	PT, CG	T_1_, T_2_, and T_3_	-Descriptive analyses -T1-T2 (Treatment Effect): ANCOVA, complete case and multiple imputation analyses, subgroup analyses, quantile regression -T2-T3 (Sustainability): ANCOVA, complete case and multiple imputation analyses, subgroup analyses
Depressive symptoms	CESD-10 [56]	PT, CG	T_1_, T_2_, and T_3_	-Descriptive analyses -T1-T2 (Treatment Effect): ANCOVA, complete case and multiple imputation analyses, quantile regression -T2-T3 (Sustainability): ANCOVA, complete case and multiple imputation analyses
Anxiety symptoms	Generalized Anxiety Disorder scale (GAD-7) [57]	PT, CG	T_1_, T_2_, and T_3_	-Descriptive analyses -T1-T2 (Treatment Effect): ANCOVA, complete case and multiple imputation analyses, quantile regression -T2-T3 (Sustainability): ANCOVA, complete case and multiple imputation analyses
Perceived social support	Personal Resource Questionnaire (PRQ-2000) [58]	PT	T_1_, T_2_, and T_3_	-Descriptive analyses -T1-T2 (Treatment Effect): ANCOVA, complete case and multiple imputation analyses, quantile regression -T2-T3 (Sustainability): ANCOVA, complete case and multiple imputation analyses
Communication between patients and providers	Patient-Provider Communication questionnaire [59]	PT, CG	T_1_ and T_2_	-Descriptive analyses -T1: z test of proportions for group comparison, complete case analysis -T2: z test of proportions for group comparison, complete case analysis
Client-centredness of care	Adapted Client-Centred Care Questionnaire (CCCQ) [60]	PT, CG	T_1_, T_2_, and T_3_	-Descriptive analyses -T1: z test of proportions for group comparison, complete case analysis -T2: z test of proportions for group comparison, complete case analysis
Care experience	Integrated Care Patient-Reported Experience Measures (IC-PREMs) [61]	PT, CG	T_1_, and T_2_	-Descriptive analyses -T1: z test of proportions for group comparison, complete case analysis -T2: z test of proportions for group comparison, complete case analysis
Unmet care needs	Unmet Care Needs questionnaire	PT, CG	T_1_, T_2_, and T_3_	-Descriptive analyses - T1: z test of proportions for group comparison, complete case analysis -T2: z test of proportions for group comparison, complete case analysis
Caregiver strain	Modified Caregiver Strain Index (CSI) [62]	CG	T_1_, T_2_, and T_3_	-Descriptive analyses -T1-T2 (Treatment Effect): ANCOVA, complete case analysis -T2-T3 (Sustainability): ANCOVA, complete case analysis
Utilization of health and social services and associated costs	Institute for Clinical Evaluative Sciences (IC/ES), Health and Social Services Utilization Inventory (HSSUI) [63, 64]	PT, CG	T_1_, T_2_, and T_3_	-Descriptive analyses -T1-T2 (Treatment Effect): Mann-Whitney U test, complete case analysis, Hazard Ratio (95% CI) & Risk Difference (95% CI) for 30, 90 &180-day ED visits and hospital admissions -T2-T3 (Sustainability): Mann-Whitney U test, complete case analysis

*PT = patient; CG = caregiver, *T1 = baseline; T2 = 6-month; T3 = 12 months

##### Older adult participant measures

The primary measure of effectiveness is mental functioning of the older adult participants as measured by the mental component score (MCS) of the Veterans Rand 12-item health survey (VR-12) [55]. Secondary outcome measures include: the physical component score (PCS) of the VR-12 [55] to measure changes in physical functioning; the Centre for Epidemiologic Studies Depression Scale (CESD-10) [56] to determine the presence and severity of depressive symptoms; the Generalized Anxiety Disorder Scale (GAD-7) [57] to determine the presence and severity of anxiety; the Personal Resource Questionnaire (PRQ-2000) Part Two [58] to measure perceived social support; and a subset of questions from the Patient Provider Communication Questionnaire (PPC) [59], the Client Centered Care Questionnaire (CCCQ) [60], the Intermediate Care for Older People Home-Based-Integrated Care Patient-Reported Experience Measures (IC-PREMs) [61], and an Unmet Care Needs questionnaire developed for the purposes of this study to measure patient experience. A complete copy of the questionnaire that includes these measures is provided as supplementary data (see Additional file 1).

##### Caregiver measures

Caregivers of older adult participants will be assessed for caregiver strain using the Modified Caregiver Strain Index (CSI) [62]. Other caregiver outcome measures include: the CESD-10 [56] to measure the presence and severity of depressive symptoms, the GAD-7 [57] to measure the presence and severity of anxiety, the mental and physical component score of the VR-12 [55] to measure health-related quality of life; and a subset of questions from the PPC [59], the CCCQ [60], the IC-PREMs [61], and the Unmet Care Needs questionnaire developed for the purposes of this study to measure caregiver experience. A complete copy of the questionnaire that includes these measures is provided as supplementary data (see Additional file 2).

##### Cost of use of health and social services

The study will link participants to Institute for Clinical Evaluative Sciences (IC/ES) administrative databases to identify the use of emergency room visits, hospital admissions, use of family physicians, physician specialists, and home care services, and transfers to long-term care. In addition, the Health and Social Services Utilization Inventory (HSSUI) [63] will be used to measure the utilization of health and social services as reported by study participants and caregivers over the duration of the study. The HSSUI consists of questions about the respondent’s use of six categories of direct healthcare services: a) primary care; b) emergency department and specialists; c) hospital days; d) seven types of other health and social professionals; e) prescribed medications; and f) lab services. The data from the HSSUI will be used in combination with the IC/ES administrative data as a back-up in case we are unable to obtain IC/ES data at all three timepoints.

The six-month cost data will be derived from the product of “quantity” data reported on the HSSUI and 2015–2016 “price” data obtained by our team for the HSSUI [64]. The costs of use of health and social services will include the costs associated with delivery of the nurse-led intervention. This will form the basis of the cost-analysis, from a societal perspective that will compare changes in the costs of use of health and social services between participants in the intervention and control groups from baseline to 6- months, and from 6-months to 12-months post intervention. A complete copy of the HSSUI questionnaire is included as supplementary data (see Additional files 1 and 2). Table 1 provides an overview of the effectiveness evaluation, including the outcome, measures, group, and tiepoint for data collection.

#### Analysis: effectiveness evaluation

Descriptive statistics will be used to summarize outcome values at baseline, 6 months and 12 months. Means and standard deviations will be used for continuous outcomes, and frequency and percentages will be used for categorical outcomes. Intention-to-treat analyses using analysis of covariance (ANCOVA) will be used to assess group differences in continuous outcomes from baseline to T2 in order to determine if the intervention was effective over the intervention period. The outcomes include: PRQ-2000, CESD-10, GAD-7, VR-12 PCS, VR-12 MCS, the PPC, CCCQ, and the IC-PREMs. The ANCOVA model will use the 6-month outcome value as the dependent variable, the group indicator as the independent variable and the baseline outcome value as the covariate. Model results will be expressed as mean group differences with accompanying 95% confidence limits. Quantile regression will also be used to examine the treatment effect over a broader range of values (beyond the mean) for the outcome variables. Assessment of group differences in continuous outcomes from T_2_ to T_3_ will be assessed using ANCOVA and will be aimed at assessing the sustainability of the intervention effects (see Table 1).

Z tests of proportions and McNemar tests will be used to assess changes within each group in the number of participants with acute care episodes and the number of acute care episodes from baseline to T_2_, and from T_2_ to T_3_ Acute care episodes include emergency department visits and hospital admissions. Risk difference (intervention risk – control risk) and accompanying 95% confidence limits will be calculated for 6 months for emergency department visits and hospital readmissions for 6 months. As was noted earlier, acute care episodes will be identified using administrative data from IC/ES, and self-reported data from the HSSUI. Due to the highly skewed nature of cost data, a non-parametric test will be used to compare the change in health and social service use and costs from baseline to T_2_ and from T_2_ to T_3_ for the two groups. Outliers are particularly common for certain healthcare costs such as hospital admissions, thus we will conduct a sensitivity analysis to assess the impact of outliers on the cost comparison.

Subgroup analyses will be conducted for the VR-12 outcomes to determine if the intervention is effective for subgroups of patients from baseline to T2. The following baseline variables have been selected a priori for testing subgroup effects: age, sex, number of chronic conditions, depressive symptoms (CESD-10), site, and dose of the intervention (number of home visits and telephone contacts). Multiple imputation will be used to address missing data. All data analyses will assume a level of significance of 0.05 and be performed using SAS Version 9.4.

#### Implementation evaluation: data collection methods, management and analysis

As noted previously, the CAST trial is a Type II hybrid effectiveness implementation study, with equal weight assigned to assessing program effectiveness and implementation. Examining the implementation of interventions is important as these data can provide an understanding of the conditions under which an intervention can be successfully implemented [65]. The implementation evaluation will include three areas of focus: 1) assessing implementation outcomes; 2) identifying how the care transition intervention was adapted and implemented in each setting; and 3) gathering information on what is required to sustain and scale-up the intervention.

Peters and colleagues [66] indicate that assessing implementation outcomes (such as adoption, acceptability and feasibility) can provide insights into the success of an intervention’s implementation. A subset of implementation outcomes identified by Peters et al. [66] will be examined in this study, specifically adoption, acceptance, feasibility, and fidelity The second two components of the implementation evaluation (implementation and sustainability of the intervention) will be guided by the Consolidated Framework for Implementation Research (CFIR) [65]. CFIR provides an organizational framework for implementation science research. It includes five key domains: i) intervention characteristics – this includes the elements of an intervention that may influence implementation (such as complexity, adaptability and cost); ii) outer setting – this includes external factors that may influence the implementation of an intervention, including political, social and economic factors; iii) inner setting – the internal organizational realities that may influence implementation (e.g., organizational structures, culture, and implementation readiness); iv) characteristics of individuals involved in the intervention, the organization, and intervention recipients; and v) implementation process including how the implementation was planned and executed, and the level of engagement of various actors [65].

#### Implementation measures and data sources

##### Implementation outcomes

Five implementation outcomes will be examined as part of this study: i) adoption or uptake refers to the engagement rate and the dose of the intervention by research participants defined as the number of home visits and telephone contacts during the six-month intervention; ii) acceptance refers to the acceptability of the intervention by study participants assessed using semi-structured interviews with participants; iii) feasibility refers to the ability to recruit participants who meet the eligibility criteria, and the feasibility of delivering the intervention in the local setting; and iv) fidelity is the extent to which the intervention was implemented as intended (see Table 2). Reasons for discontinuing the intervention will be recorded. The interview guide for the older adult and caregiver participants receiving the CAST intervention is provided as supplementary data (see Additional file 3).

**Table 2:** CAST Study Implementation Evaluation Summary

Question/Focus	Definition/Data Source	Method of Analysis
Implementation outcomes [66] a) Adoption (uptake) b) Acceptability c) Feasibility d) Fidelity	a) Engagement rate b) Post-intervention interviews; care transition coordinator direct and indirect activities c) Recruitment rate d) Fidelity checklist)	a) Proportion of participants who agreed to at least one CTC visit among those in the intervention group Calculate the dose of the intervention, defined as the number of CTC home visits and telephone contacts during the six-month intervention b) Qualitative content analysis c) Proportion of eligible individuals who enrolled in the study d) Mean number of times each core component of the intervention was implemented
Implementation and adaptation of CAST intervention	• Notes from meetings with sites • Research team meeting notes • Research coordinator notes • Care transition Coordinator interviews • Notes from CAB meetings and focus groups	The Consolidated Framework for Implementation Research (CFIR) [65, 67] is being used to guide the development of interview guides and will serve as a framework for data coding and analysis. Content analysis [68] will be conducted using primarily deductive approach.
Sustainability and scale-up	• Meeting notes with sites • Care Transition Coordinator interviews • Notes from CAB and focus groups • Research Coordinator notes • Research team meeting notes • Citizen panel	Qualitative content analysis [68] will be used to identify themes related to the sustainability as well as scale-up of the CAST intervention.

##### Adaptation and implementation of the intervention

A variety of data sources will be used to examine how the intervention was implemented and adapted in each of the three sites. This will include: minutes/notes from meetings with each of the sites (conducted prior to and during the implementation of the intervention); research team meeting notes (over the course of the study); CTC interview transcripts (two interviews will be conducted with each CTC, the first after engaging with the first 3–4 intervention participants and the second towards the end of the intervention period); notes made by the study coordinator (over the course of the study); notes from Community Advisory Board (CAB) meetings (six meetings will be held); and CAB focus group transcripts (two focus groups will be conducted with each CAB, the first after the third CAB meeting and the second after the last CAB meeting) (see Table 2). The interview guide for the CTCs is provided as supplementary data (see Additional file 4). The interview guide for the CAB focus group is provided as supplementary date (see Additional file 5).

##### Sustainability and scale-up

Examining the potential for sustainability and scale-up will involve the data sources described in Table 2. In addition, notes and materials from a Citizen Panel will also contribute to this aspect of the implementation evaluation. A Citizen Panel is an engagement process involving individuals with lived experience who come together to review existing evidence and develop recommendations based on a question of interest (https://www.mcmasterforum.org/spark-action/citizen-panels). We will be working with the McMaster Health Forum (https://www.mcmasterforum.org/) to host a Citizen Panel to examine how best to engage older adults and family care partners in improving transitional care. Table 2 provides an overview of the implementation evaluation, including the areas of focus, data sources and method of analysis.

#### Analysis: implementation evaluation

##### Quantitative data

Descriptive statistics will be used to summarize the Adoption (engagement rate, the dose of the intervention), and the level of fidelity to treatment for each core component of the intervention at 6 months. Means and standard deviations will be used for continuous outcomes, and frequency and percentages will be used for categorical outcomes. Cost analysis was described in the previous section.

##### Qualitative data

Interview and focus group data will be transcribed verbatim, and Research Coordinator and meetings notes will be typed up. All data will then be uploaded into NVivo 12 (QSR). For each data source (i.e., CTC meeting notes, CAB focus groups), two notes/transcripts will be selected and independently coded by two members of the research team. CFIR [65] will be used as a framework for coding. The two researchers will meet to develop the initial coding structure for each data source; each of the remaining notes/transcripts within that source will be coded using the coding framework. To promote consistency in coding across data sources, researchers will code more than one type of data source. Meetings with all researchers involved in coding will be held during the analysis period to share coding frameworks as well as interim codes and themes. The team of researchers will work to develop the final agreed upon themes.

#### Project Management

The Cabassa et al’s Collaborative Intervention Planning Framework [69] will be used to achieve collective impact [70] by establishing a variety of formal governing structures, including a Research Steering Committee, Community Advisory Boards, and a Patient and Caregiver Ad Hoc Group. The Collaborative Intervention Planning Framework involves collaborations between stakeholders and researchers to tailor health interventions. It draws upon the principles of community-based participatory research and intervention mapping [69]. Collective Impact involves the collaboration of stakeholders across sectors working to address complex problems [71]. It involves developing a shared vision of the issue and the intervention to address the issue; agreement on evaluation measures; open, ongoing communication; and foundational support for ongoing collaboration. A key component of this patient-oriented research project is the meaningful engagement of patients (including family caregivers) who reflect the population of interest in all stages of the research process. This includes assisting with: the identification of the research priorities and questions, designing the CAST intervention, the implementation of research studies, the interpretation of the results, and participating in knowledge dissemination [71].

The Research Steering Committee consists of the co-Principal Investigators (MMR and CM) who are responsible for overseeing all aspects of the study; the co-investigator (RG) overseeing the patient engagement component of the study; the research coordinator (AB) who coordinates the implementation of the study and supports the sites, and the four co-investigators (DU, AG, JM, CW) overseeing the research sites. The Research Steering Committee is supported by the Aging, Community and Health Research Unit [72] as well as the broader research team. The Committee will meet weekly or biweekly and draw on the expertise of other investigators as needed.

In collaboration with each community, local Community Advisory Boards (CABs) will be established. The role of the CABs is to oversee the implementation of the intervention in each community, problem-solve challenges that arise related to implementation (e.g., challenges with recruitment, identify health and social care resources in the community to support the intervention RN with implementation care delivery, and advise the research team on how to adapt the intervention to the local setting. CAB membership will include older adult patients, caregivers, and other members of the public; key health and social care providers involved in transitional care (e.g., representatives from hospitals, home care services, community support services); and representatives from the research team. Because each community is unique, the specific organizations represented within each CAB will vary from site to site. To support the involvement of patients, caregivers and members of the public, and to demonstrate their value to the process, these individuals will receive $75 stipend for each meeting attended. It is anticipated that the CABs will meet six times over the course of the study.

The Patient and Caregiver Ad Hoc group will be comprised of patient and caregiver co-researchers and supported by the RC and the co-investigator leading the patient engagement component of the study. The Patient and Caregiver Ad Hoc group will meet as needed during the course of the study to bring the patient/caregiver perspective to research-related issues, such as the selection of patient-relevant outcomes, review of study materials (e.g., consent forms, interview guides), interpretation of study findings, and knowledge dissemination. Like patient and caregiver representatives on the CABs, members of the Patient and Caregiver Ad Hoc group will receive a $75 stipend for each meeting/consultation.

#### Ethics and dissemination

The study is being conducted in accordance with the Tri-Council Policy Statement, *Ethical Conduct for Research Involving Humans* [73]. Institutional ethics approval was obtained from: the McMaster University Hamilton Integrated Research Ethics Board (REB) (# 2586); the Office of Research Ethics at the University of Waterloo (#40867); the Laurentian University REB (#6009840), and the REBs from the study sites (Health Sciences North REB # 17–007; Joseph Brant Hospital REB #000–039-17). Ethics approval will be renewed on an annual basis as required for the duration of the study. Written informed consent will be obtained from participants by the RA before study enrolment. Any protocol modifications will be reported to the REBs of each site. In accordance with PHIPA requirements, all electronic files with identifiable information will be stored on a password protected computer on a secure network and access will be limited to the study team. Paper files with identifiable information will be kept in a locked cabinet within a locked office. The list linking the participant’s study ID with their name will be kept secure in a locked filing cabinet within a locked office, separate from the destroyed at the end of the study.

#### Communication and dissemination of CAST trial results

Our plans to share the results of the CAST trial will be guided by the Knowledge to Action (KTA) framework [74], engaging patients, caregivers, and health and social care providers in all stages of the project [72]. The framework will address four questions: (1) What are the outputs of the research?, (2) Who are the potential users of the research outputs?, (3) What are the most effective ways to interact with these users?, and (4) How do we facilitate uptake and usability of the research outputs for the target audiences? The engagement of knowledge users is an integral part of the study, including the establishment of site-specific CABs as well as the Patient and Caregiver Ad Hoc Group. The ongoing engagement of knowledge users will help to support implementation and foster the uptake of the intervention [74].

End-of-grant knowledge translation activities will ensure that the results and project outputs are made available to those who need them and packaged in a way to support knowledge translation. Several traditional and non-traditional knowledge translation activities will be used. First, a Citizen Panel will be held to explore how best to engage older adults and family caregivers in transitional care. The dialogue generated at this event will integrate study findings with other research on care transitions. Evaluations indicate that the Citizen Panel’s brief dialogue approach is associated with strong intentions to incorporate learnings into action [75]. Second, we will host a webinar to share project findings with participating organizations across the three sites. Third, we will produce a series of traditional academic knowledge products – including publications, policy briefs, short reports, and conference presentations at scientific meetings. Finally, project information and activities will be posted on the Aging, Community and Health Research Unit website (https://achru.mcmaster.ca/ and distributed using social media channels. In collaboration with our knowledge users and partner networks, existing newsletters and websites will also be utilized to share project findings.

## Discussion

This paper described the design of pragmatic effectiveness-implementation trial of a nurse-led intervention (CAST) for older adults with MCC and depressive symptoms transitioning from hospital-to-home. The study will make four important contributions to the existing knowledge base. First, the study will assess both the implementation and effects of this transitional care intervention. Instead of focusing solely on whether the intervention ‘works’, the implementation evaluation results will be used to evaluate the sustainability of the intervention within each study site, and the potential for scalability to other jurisdictions. There is very little evidence regarding why certain transitional care interventions work in some settings or contexts but not in others [76]. This approach enables a focus on the complexity of interactions among the many contextual, macro-level factors that can affect implementation, which have been noted as highly relevant in determining both the outcomes and scalability of transitional care interventions [8, 28, 29]. Second, the study will include a cost analysis from a societal perspective – an outcome that has been overlooked in previous transitional care studies. This information will provide policy makers with a greater understanding of the ‘real world’ factors affecting implementation as well as the economic feasibility of integrating the intervention into usual care practice. Third, the study’s focus on older adults with MCC and depressive symptoms, a complex at-risk population that has been under-represented in transitional care evaluations, will enhance the generalizability of study results to this population. Fourth, the engagement of patients and caregivers in all aspects of the research, including the selection of patient- and caregiver-reported outcomes can inform future studies on outcomes that are important to assess from the perspective of patients and caregivers.

There are a few challenges associated with this project. The first area of challenge relates to recruitment and retention. Recruitment of complex, vulnerable individuals, such as older adults with depressive symptoms and MCC, has been challenging in our previous research [18]. To address recruitment barriers, we will hire, and train dedicated recruiters at each hospital site and develop a clear protocol for contacting potential study participants prior to hospital discharge. We will use clear but simple communication of study procedures, risks and potential benefits, and give potential participants the time needed to decide if they want to enrol in the study. We pilot tested the recruitment materials and informed consent forms with older adults to enhance the clarity of these materials and reduce participant burden. To enhance retention, we will attempt to ensure continuity of RAs across the three time points for data collection (baseline, 6-months, 12-months). The Research Coordinator will use a participant-tracking plan, and the RAs will maintain between-assessment contacts with participants. Interview reminder cards will be left with participants at the end of T_1_ (baseline) and T_2_ interviews. In addition, participants will receive reminder telephone calls from the Research Assistants (1 month before the T_2_ interview and 1–3 days prior to the appointment date) and reminder letters from the Research Coordinator (3 months from baseline and T_2_ interviews).

The second challenge relates to the need to adapt the intervention to the local context. Interventions are frequently modified during the implementation process to address differences between the context in which the intervention was originally designed and tested, and the one into which it is ultimately implemented. Adaptations are also expected to address differences in individual patient needs, which will result in variation in the intervention components and dose to which participants are exposed [77]. But these adaptations carry the risk of variability in the delivery of the intervention’s component features. This is an inherent limitation of pragmatic trials [78, 79], and is also a common challenge associated with transitional care evaluations. Variation in the nature and scope of interventions across settings has led authors of multiple systematic reviews to qualify their conclusions about the efficacy of transitional care models [27–29, 35]. Close monitoring throughout the duration of the study will assist the investigators in determining any adaptations that need to be made to the intervention. The research team will track any modifications made to the CAST intervention during its implementation. This information will be used to guide future refinements and possibly prepare for future research that tests the intervention on a larger scale. Such an understanding will allow stakeholders to make more informed decisions about whether and how to modify the interventions when implementing them in contexts that differ from those in which they were originally developed and tested.

The third challenge relates to the scalability and sustainability of the intervention. Although the process of expanding and scaling up a program should wait until the program shows desired results, planning for scale-up began at the beginning of the project. The intervention has been designed with scale-up in mind, with the goal of developing a plan that is a systematic, detailed strategy for large-scale implementation of the intervention in each study site. The WHO (2011) checklist [80] was used to ensure that features known to enhance the potential for scale-up were incorporated into the study’s design. This includes a) engaging in a participatory process with key stakeholders (e.g., patients, providers, policymakers); b) nurturing political commitment and program champions; and c) accumulating evidence from diverse settings and populations. These features are consistent with the Collective Impact framework [70, 71].

There are several strengths associated with the design of the study. First, as was noted previously, the use of a pragmatic RCT design optimizes applicability of the intervention to real-world practice by recruiting participants representative of the population presenting in clinical practice, offering flexible delivery of the intervention by providers, use of existing staff in practice, and the use of intention-to-treat analysis [38, 39]. Second, the focus on evaluating both the effectiveness and implementation of the intervention, which follows the type 2 hybrid effectiveness-implementation study design typology, offers the potential to expedite research into routine practice, compared to the more traditional approach of incremental implementation following evidence of the effectiveness of an intervention alone. Third, engaging patients, caregivers and members of the public in all aspects of the research is a strength that is a core component of patient-oriented research [81]. The importance of engaging patients (including caregivers and other member of the public) in patient-oriented research has been increasingly recognized in the research community [82]. In our study, patients, caregivers and members of the public will be engaged in a number of ways including serving as co-investigators identifying patient- and caregiver-relevant outcomes, advising on the implementation of CAST in the study settings, and developing recommendations for engaging older adults and caregivers in future transitional care activities.

Fourth, objective, reliable and valid measures will be used to assess a variety of patient-relevant outcomes, addressing a gap in the current literature that focuses predominantly on hospital readmissions as an outcome. Fifth, the study will evaluate the sustainability of the intervention effects by measuring the effectiveness of the intervention 6-months following completion of the intervention. Sixth, intervention fidelity will be enhanced by multiple approaches. Notably, the CTCs will be provided training, a standardized training manual, and regular meetings held with the research team throughout the intervention period to enhance the fidelity of intervention implementation. Lastly, the intervention will be evaluated in multiple sites and in diverse communities, which will enhance the generalizability of the results to other settings, as it reflects the diversity of the target population.

Conversely, there are some limitations to the study that warrant acknowledgement. First, sampling bias may influence the results, as those who volunteer to participate may be more likely to be receptive to the intervention. We will assess the extent of non-response bias by comparing the characteristics of study participants to those who decline participation in the study on their characteristics at baseline. Second, the use of a proxy respondent as a source of data for the study participants with limitations in cognition, physical health or language, may result in either an overestimation or an underestimation of the results.

The CAST intervention has the potential to inform policy decisions concerning the provision of transitional care for older adults with MCC and depressive symptoms transitioning from hospital to home. Given the rapid increase in the number of older adults living in the community, the challenges of meeting the complex care needs of older adults with MCC and depressive symptoms have the potential to place extensive burdens on a health care system that is already under strain. In Ontario, the jurisdiction where CAST is being evaluated, a recent expert advisory panel on health care reform identified a number of system-level issues the CAST study has the potential to mitigate, including an increase in the number of patients with complex needs, difficulties with system navigation, challenges transitioning between services, and the need for more effective coordination at point of care [83]. Moreover, the level of patient support offered by the CAST intervention has the potential to enable greater independence and aging at home, which is the expressed preference of community-dwelling older adults [3, 84, 85]. This project will have meaningful results for patients as the outcomes will address not only their unmet needs but reflect issues important to them. Further, the study results will support the continued development of our patient- and community-centred research agenda [72, 82]. The use of a pragmatic effectiveness-implementation trial will enhance the relevance of the results for practitioners and policy makers, thereby reducing the research-practice gap and enhance the sustainability and scalability of the intervention.

## Supplementary information

**Additional file 1.** Questionnaire to measure the effectiveness of the CAST intervention on older adults. This file includes the questionnaire that was used to measure the effectiveness of the CAST intervention on older adult outcomes from baseline to 6- and 12-months.

**Additional file 2.** Questionnaire to measure the effectiveness of the CAST intervention on caregivers. This file includes the questionnaire that was used to measure the effectiveness of the CAST intervention on caregiver outcomes from baseline to 60 and 12-months.

**Additional file 3.** Interview guide for older adult and caregiver participants receiving the CAST intervention. This file includes the interview guide that was developed to guide the semi-structured interviews with older adult and caregiver participants receiving the CAST intervention.

**Additional file 4.** Interview guide for Care Transition Coordinators. This file includes the interview guide that was developed to guide the semi-structured interviews with the Care Transition Coordinators who delivered the CAST intervention.

**Additional file 5.** Interview guide for Community Advisory Boards. This file includes the interview guide that was developed to guide the semi-structured interviews with the Community Advisory Boards.

## Data Availability

The data for this research consists of questionnaires, in-person interview transcripts and notes. Raw data cannot be publicly released due to the risk of compromising participant confidentiality.

## Abbreviations

ACHRU: Aging, Community and Health Research Unit (McMaster University, School of Nursing)
ACHRU-CAST: Aging, Community and Health Research Unit - Community Assets Supporting Transitions
MCC: Multiple chronic conditions
HRQoL: Health related quality of life
IP: Interprofessional
RN: Registered Nurse
CTC: Care Transition Coordinator
RCT: Randomized controlled trial
MCS: Mental Health Component Summary of Veterans Rand 12 Item Health Survey
PCS: Physical
PHQ-2: Patient Health Questionnaire (2 items)
CAST: Community Assets Supporting Transitions
LHIN: Local Health Integration Network
HNHB: Hamilton Niagara Haldimand Brant
NE: North East
CCAC: Community Care Access Centre
IC-PREMs: Integrated Care Patient-Reported Experience Measures
CCCQ: Client-Centred Care Questionnaire
HSSUI: Health and Social Services Utilization Inventory
CSI: Modified Caregiver Strain Index
GAD-7: Generalized Anxiety Disorder (7 items)
VR-12: Veterans Rand 12 Item Health Survey
CESD-10: Center for Epidemiological Studies on Depression (10 items)
