# Supplementary material for: Study protocol for a hospital-to-home transitional care intervention for older adults with multiple chronic conditions and depressive symptoms: a pragmatic effectiveness-implementation trial
Source: BMC Geriatr. 2020 Jul 10;20:240. doi: 10.1186/s12877-020-01638-0 (PMC7350576; doi:10.1186/s12877-020-01638-0)
Supplement: Supplementary file 1 — Additional file 1. Questionnaire to measure the effectiveness of the CAST intervention on older adults. This file includes the questionnaire that was used to measure the effectiveness of the CAST intervention on older adult outcomes from baseline to 6- and 12-months. [file 12877_2020_1638_MOESM1_ESM.docx]

**Additional file 1**

**Questionnaire to measure the effectiveness of the CAST intervention on Older Adults**

**Short Portable Mental Status Questionnaire (SPMSQ**)

| **Correct** | **Not correct** | **Questions** | **Instructions** |
| --- | --- | --- | --- |
| ❑_1_ | ❑_2_ | SP-1. What is the date today?  ______________________________________ | Correct only when the month, date, and year are all correct. |
| ❑_1_ | ❑_2_ | SP-2. What day of the week is it?  ______________________________________ | Correct only when the day is correct. |
| ❑_1_ | ❑_2_ | SP-3. What is the name of this place?  ______________________________________ | Correct if any of the description of the location is given. "My home," the correct city/town, or the correct name of the hospital/institution are all acceptable. |
| ❑_1_ | ❑_2_ | SP-4a. What is your telephone number?  ______________________________________ | Correct when the number can be verified, or the participant can repeat the same number at a later time in the interview. |
| ❑_1_ | ❑_2_ | SP-4b. What is your street address?  ______________________________________ | **Ask only if the participant does not have a telephone.** |
| ❑_1_ | ❑_2_ | SP-5. How old are you?  ______________________________________ | Correct when the stated age corresponds to the date of birth. |
| ❑_1_ | ❑_2_ | SP-6. When were you born?  ______________________________________ | Correct only when the month, date, and year are correct. |
| ❑_1_ | ❑_2_ | SP-7. Who is the prime minister of Canada now?  ______________________________________ | Requires only the correct last name. |
| ❑_1_ | ❑_2_ | SP-8. Who was the prime minister just before him?_________________________________ | Requires only the correct last name. |
| ❑_1_ | ❑_2_ | SP-9. What was your mother's maiden name?  ______________________________________ | Needs no verification; it only requires a female first name plus a last name other than the subjects. |
| ❑_1_ | ❑_2_ | SP-10. Subtract 3 from 20 and keep subtracting 3 from each new number, all the way down. ____________________________ _____________________________________ | The entire series must be performed correctly to be scored as correct. Any error in the series - or an unwillingness to attempt the series - is scored as incorrect. |
| Total CORRECT Responses: ­­­­­________ Total ERRORS: _______ | | |  |

**Sociodemographic and Health Questionnaire**

SH-1. How do you describe your gender?

❑_1_Male

❑_2_Female

❑_3_ Other (please specify): __________________________

❑_4_ Prefer not to answer

SH-2. What is your date of birth (dd/mmm/yyyy)? ______/_______ /________

SH-3. What type of accommodation do you live in?

❑_1_ House

❑_2_ Apartment

❑_3_ Retirement home

❑_4_ Supportive housing (live in building where assistance is available for activities of daily living)

❑_5_ Convalescent (short term) nursing home

❑_6_ Condominium

❑_7_ Other (please specify): __________________________

SH-4. What is your current relationship status?

❑_1_ Never married

❑_2_ Married/living with a partner

❑_3_ Widowed

❑_4_ Separated

❑_5_ Divorced/annulled

SH-5. What is the highest level of education that you have completed?

❑_1_ No schooling

❑_2_ Elementary school (8^th^ grade/less)

❑_3_ Did not complete secondary or high school

❑_4_ Completed secondary school or high school

❑_5_ Had some university/college education

❑_6_ Completed a community college, technical college, or post-secondary program (e.g. trade, technical or vocational school, CEGEP)

❑_7_ Completed a bachelor’s degree (e.g. B.A., B.Sc., B.S.N.)

❑_8_ Completed a graduate degree or professional degree (e.g. MD, DDS, DMD, DVM, OD, Masters, PhD)

SH-6. Please provide your **current** employment status by choosing the main option that applies to you.

❑_1_ Employed full-time (including self-employed or on a work training program; 30 or more hours each week)

❑_2_ Employed part-time (including self-employed or on a work training program; under 30 hours each week)

❑_3_ Unemployed and looking for work

❑_4_ At school or in full-time education

❑_5_ Unable to work due to a long-term sickness or disability

❑_6_ Looking after your home/family

❑_7_ Retired from paid work

❑_8_ Doing something else

| SH-7. Please estimate in which of the following groups your total annual household income falls? | | |
| --- | --- | --- |
| ❑_1_ Less than $5,000 | ❑_6_ $30,000 to less than $40,000 | ❑_11_ $80,000 to less than $90,000 |
| ❑_2_ $5,000 to less than $10,000 | ❑_7_ $40,000 to less than $50,000 | ❑_12_ $90,000 to less than $100,000 |
| ❑_3_ $10,000 to less than $15,000 | ❑_8_ $50,000 to less than $60,000 | ❑_13_ $100,000 to less than $150,000 |
| ❑_4_ $15,000 to less than $20,000 | ❑_9_ $60,000 to less than $70,000 | ❑_14_ $150,000 and over |
| ❑_5_ $20,000 to less than $30,000 | ❑_10_ $70,000 to less than $80,000 | ❑_15_ Prefer not to answer |

SH-8. Are you

❑_1_ Canadian Citizen

❑_2_ Indigenous (First Nations, Inuit, or Métis)

❑_3_ Landed immigrant

❑_4_ Other (please specify): ___________________________

SH-9. a) What is your ethnic background? (select all that apply)

❑_1_ African

❑_2_ Asian (Chinese, Japanese, Korean, etc.)

❑_3_ Caribbean

❑_4_ European

❑_5_ Latin, Central or South American

❑_6_ Indigenous (First Nations, Inuit, or Métis)

❑_7_ South Asian (East Indian, Pakistani, Sri-Lankan, etc.)

❑_8_ Southeast Asian (Filipino, Cambodian, Laotian, Malaysian, Vietnamese, etc.)

❑_9_ West Asian or Middle Eastern (Afghan, Iranian, etc.)

❑_10_ Other (please specify): ___________________________

b) If Indigenous:

❑_1_ First Nations

❑_2_ Métis

❑_3_ Inuk

❑_4_ Other (please specify): ___________________________

SH-10. What language(s) do you usually speak at home? Select as many as apply.

❑_1_ English

❑_2_ French

❑_3_ Other language (please specify): ___________________________

SH-11. Do you currently live with someone? (Select all that apply)

❑_1_ Live alone

❑_2_ Spouse or partner

❑_3_ Friend(s)

❑_4_ Live in group setting with non-relative (e.g., retirement home)

❑_5_ Children

❑_6_ Family members (please specify):

❑_7_ Other (please specify):

**Personal Resource Questionnaire**

PRQ-1. There is someone I feel close to who makes me feel secure.

❑_1_ Strongly disagree

❑_2_ Disagree

❑_3_ Somewhat disagree

❑_4_ Neutral

❑_5_ Somewhat agree

❑_6_ Agree

❑_7_ Strongly agree

PRQ-2. I belong to a group in which I feel important.

❑_1_ Strongly disagree

❑_2_ Disagree

❑_3_ Somewhat disagree

❑_4_ Neutral

❑_5_ Somewhat agree

❑_6_ Agree

❑_7_ Strongly agree

PRQ-3. People let me know what I do well at my daily responsibilities (job, homemaking).

❑_1_ Strongly disagree

❑_2_ Disagree

❑_3_ Somewhat disagree

❑_4_ Neutral

❑_5_ Somewhat agree

❑_6_ Agree

❑_7_ Strongly agree

PRQ-4. I have enough contact with the person who makes me feel special.

❑_1_ Strongly disagree

❑_2_ Disagree

❑_3_ Somewhat disagree

❑_4_ Neutral

❑_5_ Somewhat agree

❑_6_ Agree

❑_7_ Strongly agree

PRQ-5. I spend time with others who have the same interests that I do.

❑_1_ Strongly disagree

❑_2_ Disagree

❑_3_ Somewhat disagree

❑_4_ Neutral

❑_5_ Somewhat agree

❑_6_ Agree

❑_7_ Strongly agree

PRQ-6. Others let me know that they enjoy being with me (projects).

❑_1_ Strongly disagree

❑_2_ Disagree

❑_3_ Somewhat disagree

❑_4_ Neutral

❑_5_ Somewhat agree

❑_6_ Agree

❑_7_ Strongly agree

PRQ-7. There are people who are available if I need help over an extended period of time.

❑_1_ Strongly disagree

❑_2_ Disagree

❑_3_ Somewhat disagree

❑_4_ Neutral

❑_5_ Somewhat agree

❑_6_ Agree

❑_7_ Strongly agree

PRQ-8. Among my group of friends we do favours for each other.

❑_1_ Strongly disagree

❑_2_ Disagree

❑_3_ Somewhat disagree

❑_4_ Neutral

❑_5_ Somewhat agree

❑_6_ Agree

❑_7_ Strongly agree

PRQ-9. I have the opportunity to encourage others to develop their interests and skills.

❑_1_ Strongly disagree

❑_2_ Disagree

❑_3_ Somewhat disagree

❑_4_ Neutral

❑_5_ Somewhat agree

❑_6_ Agree

❑_7_ Strongly agree

PRQ-10. I have relatives or friends that will help me out even if I can’t pay them back.

❑_1_ Strongly disagree

❑_2_ Disagree

❑_3_ Somewhat disagree

❑_4_ Neutral

❑_5_ Somewhat agree

❑_6_ Agree

❑_7_ Strongly agree

PRQ-11. When I am upset, there is someone I can be with who lets me be myself.

❑_1_ Strongly disagree

❑_2_ Disagree

❑_3_ Somewhat disagree

❑_4_ Neutral

❑_5_ Somewhat agree

❑_6_ Agree

❑_7_ Strongly agree

PRQ-12. I know that others appreciate me as a person.

❑_1_ Strongly disagree

❑_2_ Disagree

❑_3_ Somewhat disagree

❑_4_ Neutral

❑_5_ Somewhat agree

❑_6_ Agree

❑_7_ Strongly agree

PRQ-13. There is someone who loves and cares about me.

❑_1_ Strongly disagree

❑_2_ Disagree

❑_3_ Somewhat disagree

❑_4_ Neutral

❑_5_ Somewhat agree

❑_6_ Agree

❑_7_ Strongly agree

PRQ-14. I have people to share social events and fun activities with.

❑_1_ Strongly disagree

❑_2_ Disagree

❑_3_ Somewhat disagree

❑_4_ Neutral

❑_5_ Somewhat agree

❑_6_ Agree

❑_7_ Strongly agree

PRQ-15. I have a sense of being needed by another person.

❑_1_ Strongly disagree

❑_2_ Disagree

❑_3_ Somewhat disagree

❑_4_ Neutral

❑_5_ Somewhat agree

❑_6_ Agree

❑_7_ Strongly agree

**Chronic Conditions**

a) Do you have any of the following types of health problems?

| **Chronic condition** | **Yes** | **No** | **b) If yes, for 6 months or longer?** |
| --- | --- | --- | --- |
| CC-1. Hypertension (high blood pressure) | ❑_1_ | ❑_2_ | ❑_1_ Yes  ❑_2_ No |
| CC-2. Stomach problem (i.e., reflux or peptic ulcer symptoms) | ❑_1_ | ❑_2_ | ❑_1_ Yes  ❑_2_ No |
| CC-3. Depression | ❑_1_ | ❑_2_ | ❑_1_ Yes  ❑_2_ No |
| CC-4. Thyroid disorder | ❑_1_ | ❑_2_ | ❑_1_ Yes  ❑_2_ No |
| CC-5. Chronic musculoskeletal condition causing pain or limitation | ❑_1_ | ❑_2_ | ❑_1_ Yes  ❑_2_ No |
| CC-6. Osteoarthritis or other arthritis | ❑_1_ | ❑_2_ | ❑_1_ Yes  ❑_2_ No |
| CC-7. Osteoporosis | ❑_1_ | ❑_2_ | ❑_1_ Yes  ❑_2_ No |
| CC-8. Asthma or lung problem, like chronic bronchitis, emphysema, or COPD (chronic obstructive pulmonary disorder) | ❑_1_ | ❑_2_ | ❑_1_ Yes  ❑_2_ No |
| CC-9. Cancer in the past 5 years (including melanoma, but not other skin cancers) | ❑_1_ | ❑_2_ | ❑_1_ Yes  ❑_2_ No |
| CC-10. Heart failure (including heart valve disease or replacement) | ❑_1_ | ❑_2_ | ❑_1_ Yes  ❑_2_ No |
| CC-11. Hyperlipidemia (high cholesterol) | ❑_1_ | ❑_2_ | ❑_1_ Yes  ❑_2_ No |
| CC-12. Obesity | ❑_1_ | ❑_2_ | ❑_1_ Yes  ❑_2_ No |
| CC-13. Colon problem (irritable bowel, Crohn’s disease, ulcerative colitis, diverticulosis) | ❑_1_ | ❑_2_ | ❑_1_ Yes  ❑_2_ No |
| CC-14. Anxiety | ❑_1_ | ❑_2_ | ❑_1_ Yes  ❑_2_ No |
| CC-15. Diabetes | ❑_1_ | ❑_2_ | ❑_1_ Yes  ❑_2_ No |
| CC-16. Chronic liver disease (including chronic hepatitis or cirrhosis) | ❑_1_ | ❑_2_ | ❑_1_ Yes  ❑_2_ No |
| CC-17. Chronic urinary problem | ❑_1_ | ❑_2_ | ❑_1_ Yes  ❑_2_ No |
| CC-18. Chronic kidney disease or failure | ❑_1_ | ❑_2_ | ❑_1_ Yes  ❑_2_ No |
| CC-19. Cardiovascular disease (including angina, previous heart attack, atrial fibrillation, lower limbs circulation problems) | ❑_1_ | ❑_2_ | ❑_1_ Yes  ❑_2_ No |
| CC-20. Alzheimer disease or another form of dementia | ❑_1_ | ❑_2_ | ❑_1_ Yes  ❑_2_ No |
| CC-21. Stroke (cerebrovascular accident or transient ischemic attack) | ❑_1_ | ❑_2_ | ❑_1_ Yes  ❑_2_ No |
| CC-22. HIV | ❑_1_ | ❑_2_ | ❑_1_ Yes  ❑_2_ No |
| CC-23. Other (please specify): ________________________ | ❑_1_ | ❑_2_ | ❑_1_ Yes  ❑_2_ No |
| CC-24. Other (please specify): ________________________ | ❑_1_ | ❑_2_ | ❑_1_ Yes  ❑_2_ No |
| CC-25. Other (please specify): ________________________ | ❑_1_ | ❑_2_ | ❑_1_ Yes  ❑_2_ No |
| CC-26. Other (please specify): ________________________ | ❑_1_ | ❑_2_ | ❑_1_ Yes  ❑_2_ No |

CC-27. Please list any conditions that were newly diagnosed within the **past 6 months**.
__________________________________________________________________________________________

__________________________________________________________________________________________

________________________________________________________________________________________

**VR-12**

The following questions ask for your views about your health—how you feel and how well you are able to do your usual activities. There are no right or wrong answers; please choose the answer that best fits your life right now.

VR-1. In general, would you say your health is:

❑_1_ Excellent

❑_2_ Very good

❑_3_ Good

❑_4_ Fair

❑_5_ Poor

VR-2. The following questions are about activities you might do during a typical day. Does your health limit you in these activities? If so, how much?

a) **Moderate activities**, such as moving a table, pushing a vacuum cleaner, bowling or playing golf?

❑_1_ Yes, limited a lot

❑_2_ Yes, limited a little

❑_3_ No, not limited at all

b) Climbing **several** flights of stairs?

❑_1_ Yes, limited a lot

❑_2_ Yes, limited a little

❑_3_ No, not limited at all

VR-3. During the past four (4) weeks, have you had any of the following problems with your work or other regular daily activities as a result of your physical health?

a) Accomplished **less** than you would like.

❑_1_ Yes, all of the time

❑_2_ Yes, most of the time

❑_3_ Yes, some of the time

❑_4_ Yes, a little of the time

❑_5_ No, none of the time

b) Were limited in the **kind** of work or other activities.

❑_1_ Yes, all of the time

❑_2_ Yes, most of the time

❑_3_ Yes, some of the time

❑_4_ Yes, a little of the time

❑_5_ No, none of the time

VR-4 . During the past four (4) weeks, have you had any of the following problems with your work or other regular daily activities as a result of any emotional problems (such as feeling depressed or anxious)?

a) Accomplished **less** than you would like.

❑_1_ Yes, all of the time

❑_2_ Yes, most of the time

❑_3_ Yes, some of the time

❑_4_ Yes, a little of the time

❑_5_ No, none of the time

b) Didn’t do work or other activities as **carefully** as usual.

❑_1_ Yes, all of the time

❑_2_ Yes, most of the time

❑_3_ Yes, some of the time

❑_4_ Yes, a little of the time

❑_5_ No, none of the time

VR-5. During the past four (4) weeks, how much did pain interfere with your normal work (including both work outside the home and housework)?

❑_1_ Not at all

❑_2_ A little bit

❑_3_ Moderately

❑_4_ Quite a bit

❑_5_ Extremely

VR-6. These questions are about how you feel and how things have been with you during the past four (4) weeks. For each question, please give the one answer that comes closest to the way you have been feeling.

How much of the time during the past four (4) weeks:

a) Have you felt calm and peaceful?

❑_1_ All of the time

❑_2_ Most of the time

❑_3_ A good bit of the time

❑_4_ Some of the time

❑_5_ A little of the time

❑_6_ None of the time

b) Did you have a lot of energy?

❑_1_ All of the time

❑_2_ Most of the time

❑_3_ A good bit of the time

❑_4_ Some of the time

❑_5_ A little of the time

❑_6_ None of the time

c) Have you felt downhearted and blue?

❑_1_ All of the time

❑_2_ Most of the time

❑_3_ A good bit of the time

❑_4_ Some of the time

❑_5_ A little of the time

❑_6_ None of the time

During the past four (4) weeks:

VR-7 . How much of the time has your physical health or emotional problems interfered with your social activities (like visiting with friends, relatives, etc.)?

❑_1_ All of the time

❑_2_ Most of the time

❑_3_ Some of the time

❑_4_ A little of the time

❑_5_ None of the time

Now, we’d like to ask you some questions about how your health may have changed.

VR-8 . Compared to one year ago, how would you rate your physical health in general now?

❑_1_ Much better

❑_2_ Slightly better

❑_3_ About the same

❑_4_ Slightly worse

❑_5_ Much worse

VR-9. Compared to one year ago, how would you rate your emotional problems (such as feeling anxious, depressed or irritable) now?

❑_1_ Much better

❑_2_ Slightly better

❑_3_ About the same

❑_4_ Slightly worse

❑_5_ Much worse

**Centre for Epidemiological Studies Short Depression Scale (CES-D-10)**

Next, I will read a list of the ways you might have felt or behaved recently. For each of the following statements, please select the response which best describes how often you have felt this way **during the last week.**

| **During the last week:** | | **Rarely or None**  **of the Time**  **(Less than 1 Day)** | **Some or a**  **Little of the**  **Time (1-2 Days)** | **Occasionally**  **or Moderate**  **(3-4 Days)** | **Most or All**  **of the Time**  **(5-7 Days)** |
| --- | --- | --- | --- | --- | --- |
| DS-1. | I was bothered by things that usually don't bother me. | ❑_0_ | ❑_1_ | ❑_2_ | ❑_3_ |
| DS-2. | I had trouble keeping my mind on what I was doing. | ❑_0_ | ❑_1_ | ❑_2_ | ❑_3_ |
| DS-3. | I felt depressed. | ❑_0_ | ❑_1_ | ❑_2_ | ❑_3_ |
| DS-4. | I felt that everything I did was an effort. | ❑_0_ | ❑_1_ | ❑_2_ | ❑_3_ |
| DS-5. | I felt hopeful about the future. | ❑_0_ | ❑_1_ | ❑_2_ | ❑_3_ |
| DS-6. | I felt fearful. | ❑_0_ | ❑_1_ | ❑_2_ | ❑_3_ |
| DS-7. | My sleep was restless. | ❑_0_ | ❑_1_ | ❑_2_ | ❑_3_ |
| DS-8. | I was happy. | ❑_0_ | ❑_1_ | ❑_2_ | ❑_3_ |
| DS-9. | I felt lonely. | ❑_0_ | ❑_1_ | ❑_2_ | ❑_3_ |
| DS-10 | I could not get *"going."* | ❑_0_ | ❑_1_ | ❑_2_ | ❑_3_ |

**Depression History**

DH-1. a) Have you ever been diagnosed with depression?

❑_1_ Yes

❑_2_ No

b) If yes*:* In what year were you diagnosed? _______________

DH-2. Over the last 2 weeks, how often have you been bothered by thoughts that you would be better off dead or of hurting yourself in some way?

❑_1_ Not at all

❑_2_ Several days

❑_3_ More than half the days

❑_4_ Nearly every day

**Generalized Anxiety Disorder Screener (GAD-7)**

| Over the **last 2 weeks**, how often have you been bothered by the following problems? | **Not at all** | **Several days** | **More than half the days** | **Nearly every day** |
| --- | --- | --- | --- | --- |
| GAD-1. Feeling nervous, anxious, or on edge | ❑_0_ | ❑_1_ | ❑_2_ | ❑_3_ |
| GAD-2. Not being able to stop or control worrying | ❑_0_ | ❑_1_ | ❑_2_ | ❑_3_ |
| GAD-3. Worrying too much about different things | ❑_0_ | ❑_1_ | ❑_2_ | ❑_3_ |
| GAD-4. Trouble relaxing | ❑_0_ | ❑_1_ | ❑_2_ | ❑_3_ |
| GAD-5. Being so restless that it is hard to sit still | ❑_0_ | ❑_1_ | ❑_2_ | ❑_3_ |
| GAD-6. Becoming easily annoyed or irritable | ❑_0_ | ❑_1_ | ❑_2_ | ❑_3_ |
| GAD-7. Feeling afraid as if something awful might happen | ❑_0_ | ❑_1_ | ❑_2_ | ❑_3_ |

*If the participant indicated that they have been bothered by any of the above problems*:

GAD-8. You indicated that you have been bothered by *(list items above that were selected)*. How difficult have these problems made it for you to do your work, take care of things at home, or get along with other people?

❑_1_ Not difficult at all

❑_2_ Somewhat difficult

❑_3_ Very difficult

❑_4_ Extremely difficult

**Health and Social Service Utilization Inventory (HSSUI)**

**The following questions are related to the health and social services you have used in the past 6 months.**

In the **past 6 months**, have you used any of the following services (if yes for any of the below, please document how often service was used and associated costs – distinguish between those paid by private insurance, government (OHIP), cash, or any other source):

| **Service** | **Service used** | **How often used in past 6 months?**  Please show calculations for frequently used services, e.g. # of times per week x # of months. | **If you paid for any of these services yourself, please enter the cost below** |
| --- | --- | --- | --- |
| HSS-1. Family Physician |  |  |  |
| 1. Family Physician Visit:   **If yes, please specify type(s):** | ❑_1_ Yes  ❑_2_ No |  |  |
| 1. Office Visit ❑_1_ Yes |  |  | Cost/visit  $______ |
| 1. Home Visit ❑_1_ Yes |  |  | Cost/visit  $______ |
| 1. Walk-in Clinic ❑_1_ Yes |  |  | Cost/visit  $______ |
| 1. Nurse Practitioner | ❑_1_ Yes  ❑_2_ No |  | Cost/visit  $______ |
| 1. Nurse | ❑_1_ Yes  ❑_2_ No |  | Cost/visit  $______ |
| 1. Dietitian | ❑_1_ Yes  ❑_2_ No |  | Cost/visit  $______ |
| 1. Social Worker | ❑_1_ Yes  ❑_2_ No |  | Cost/visit  $______ |
| 1. Mental Health Counselor | ❑_1_ Yes  ❑_2_ No |  | Cost/visit  $______ |
| 1. Pharmacist | ❑_1_ Yes  ❑_2_ No |  | Cost/visit  $______ |
| HSS-2. Physician Specialist |  |  |  |
| 1. **If yes, please specify type(s)** i.e. geriatrician, psychiatrist, psychologist, physiatrist, urologist, gastroenterologist –if known) and name of each specialist: | ❑_1_ Yes  ❑_2_ No  Was visit  provided in home? |  |  |
|  | ❑_1_ Yes  ❑_2_ No |  | Cost/visit  $______ |
|  | ❑_1_ Yes  ❑_2_ No |  | Cost/visit  $______ |
|  | ❑_1_ Yes  ❑_2_ No |  | Cost/visit  $______ |
|  | ❑_1_ Yes  ❑_2_ No |  | Cost/visit  $______ |
|  | ❑_1_ Yes  ❑_2_ No |  | Cost/visit  $______ |
| HSS-3. Home Care Services not paid by CCAC. |  |  |  |
| 1. Personal Support Worker | ❑_1_ Yes  ❑_2_ No |  | Cost/visit  $______ |
| 1. Other, please specify: _________________________ | ❑_1_ Yes  ❑_2_ No |  | Cost/visit  $______ |
| HSS-4. Outpatient Services |  |  |  |
| 1. Homemaker | ❑_1_ Yes  ❑_2_ No |  | Cost/visit  $______ |
| 1. Optometrist | ❑_1_ Yes  ❑_2_ No |  | Cost/visit  $______ |
| 1. Delivered meal service. Include $ per meal;   inside the home or outside | ❑_1_ Yes  ❑_2_ No | # of meals:  ______ | Cost/meal  $______ |
| 1. Adult day program | ❑_1_ Yes  ❑_2_ No | # of days:  ______ | Cost/day  $______ |
| 1. Over-night respite services (e.g., long term care – not permanent placement).   **If yes,** please also note the name of facility: **____________________________________**  **____________________________________** | ❑_1_ Yes  ❑_2_ No | # of days:  ______ | Cost/day  $______ |
| 1. Social and recreational services, e.g., recreational therapy, swimming, yoga etc.   **If yes**, please name the service: ____________________________________ | ❑_1_ Yes  ❑_2_ No | # of visits:  ______ | Cost/visit  $______ |
| 1. Community Support Groups, e.g. Diabetes, Stroke, Canadian Cancer Society support group.   **If yes**, please name the group: ____________________________________ | ❑_1_ Yes  ❑_2_ No | # of groups:  ______ | Cost/group  $______ |
| 1. Transportation services (e.g. wheelchair accessible services) | ❑_1_ Yes  ❑_2_ No | # of days:  ______ | Cost/day  $______ |

| **Unmet Care Needs**  Everyone has different needs as they leave the hospital. In the next section, we will discuss some needs that others have identified. We would like to know if you’ve had a need for service support that you were not able to get.   \| UCN-1. Are the services you are receiving meeting your physical health needs? \| ❑_1_ Yes  ❑_2_ No \| **If no,** what are the reasons?  ❑_1_ Challenges with moving around  ❑_2_ Transportation difficulties  ❑_3_ Didn’t know who to call or where to go  ❑_4_ Not available in the area  ❑_5_ Not available in the time needed  ❑_6_ Wait list too long  ❑_7_ Didn’t bother/decided not to seek care  ❑_8_ No respite care  ❑_9_ Language barrier  ❑_10_ Financial barriers (e.g., service not covered by provincial/federal/personal health insurance)  ❑_11_ Ineligible for available services  ❑_12_  Other (specify)_______________________ \| \| --- \| --- \| --- \| \| UCN-2. Are the services you are receiving meeting your emotional or mental health needs? \| ❑_1_ Yes  ❑_2_ No \| **If no,** what are the reasons?  ❑_1_ Challenges with moving around  ❑_2_ Transportation difficulties  ❑_3_ Didn’t know who to call or where to go  ❑_4_ Not available in the area  ❑_5_ Not available in the time needed  ❑_6_ Wait list too long  ❑_7_ Didn’t bother/decided not to seek care  ❑_8_ No respite care  ❑_9_ Language barrier  ❑_10_ Financial barrier (e.g., service not covered by provincial/federal/personal health insurance)  ❑_11_ In eligible for available services  ❑_12_Other(specify)_______________________ \| \| UCN-3. Are the services you are receiving meeting your personal care needs (e.g., bathing, dressing)? \| ❑_1_ Yes  ❑_2_ No \| **If no,** what are the reasons?  ❑_1_ Challenges with moving around  ❑_2_ Transportation difficulties  ❑_3_ Didn’t know who to call or where to go  ❑_4_ Not available in the area  ❑_5_ Not available in the time needed  ❑_6_ Wait list too long  ❑_7_ Didn’t bother/decided not to seek care  ❑_8_ No respite care  ❑_9_ Language barrier  ❑_10_ Financial barrier (e.g., service not covered by provincial/federal/personal health insurance)  ❑_11_ Ineligible for available services  ❑_12_  Other (specify)_______________________ \| \| UCN-4. Are the services you are receiving meeting your housekeeping needs (e.g., food preparation, laundry)? \| ❑_1_ Yes  ❑_2_ No \| **If no,** what are the reasons?  ❑_1_ Challenges with moving around  ❑_2_ Transportation difficulties  ❑_3_ Didn’t know who to call or where to go  ❑_4_ Not available in the area  ❑_5_ Not available in the time needed  ❑_6_ Wait list too long  ❑_7_ Didn’t bother/decided not to seek care  ❑_8_ No respite care  ❑_9_ Language barrier  ❑_10_ Financial barrier (e.g., service not covered by provincial/federal/personal health insurance)  ❑_11_ Ineligible for available services  ❑_12_  Other (specify)_______________________ \| \| UCN-5. Are the services you are receiving meeting your home maintenance needs (e.g., snow shoveling, lawn care)? \| ❑_1_ Yes  ❑_2_ No \| **If no,** what are the reasons?  ❑_1_ Challenges with moving around  ❑_2_ Transportation difficulties  ❑_3_ Didn’t know who to call or where to go  ❑_4_ Not available in the area  ❑_5_ Not available in the time needed  ❑_6_ Wait list too long  ❑_7_ Didn’t bother/decided not to seek care  ❑_8_ No respite care  ❑_9_ Language barrier  ❑_10_ Financial barrier (e.g., service not covered by provincial/federal/personal health insurance)  ❑_11_ Ineligible for available services  ❑_12_  Other (specify)_______________________ \| \| UCN-6. Are the services you are receiving meeting your needs in terms of support with shopping for essential items (e.g., food, medicine)? \| ❑_1_ Yes  ❑_2_ No \| **If no,** what are the reasons?  ❑_1_ Challenges with moving around  ❑_2_ Transportation difficulties  ❑_3_ Didn’t know who to call or where to go  ❑_4_ Not available in the area  ❑_5_ Not available in the time needed  ❑_6_ Wait list too long  ❑_7_ Didn’t bother/decided not to seek care  ❑_8_ No respite care  ❑_9_ Language barrier  ❑_10_ Financial barrier (e.g., service not covered by provincial/federal/personal health insurance)  ❑_11_ Ineligible for available services  ❑_12_  Other (specify)_______________________ \| \| UCN-7. Are there other service needs you have had that were not met? \| ❑_1_ Yes  ❑_2_ No \| **If yes,** please specify what type of care needs were not met: ____________________________________  **W**hat are the reasons?  ❑_1_ Challenges with moving around  ❑_2_ Transportation difficulties  ❑_3_ Didn’t know who to call or where to go  ❑_4_ Not available in the area  ❑_5_ Not available in the time needed  ❑_6_ Wait list too long  ❑_7_ Didn’t bother/decided not to seek care  ❑_8_ No respite care  ❑_9_ Language barrier  ❑_10_ Financial barrier (e.g., service not covered by provincial/federal/personal health insurance)  ❑_11_ Ineligible for available services  ❑_12_  Other (specify)_______________________ \|   **EMERGENCY VISITS AND HOSPITALIZATIONS (in the past 6 months)?**  HSS-5. a) Have you used 911 in the **past 6 months**?  ❑_1_ Yes  ❑_2_ No   1. If yes: How many times? ________   HSS-6.a) Have you used ambulance services in the **past 6 months**?  ❑_1_ Yes  ❑_2_ No   1. If yes: How many times? ________   HSS-7. a) Have you visited the emergency room in the **past 6 months**?  ❑_1_ Yes  ❑_2_ No   1. If yes: How many times? ________   HSS-8. a) Have you been admitted to the hospital in the **past 6 months**?  ❑_1_ Yes  ❑_2_ No   1. If yes: How many hospital admissions in the **past 6 months**? ________ 2. Total number of days admitted within the **past 6 months**: ________ |  |
| --- | --- | --- | --- | --- | --- | --- | --- | --- | --- | --- | --- | --- | --- | --- | --- | --- | --- | --- | --- | --- | --- | --- |
|  | |

| HSS-9. a) Have you taken any **prescription medications** in the **past 2 days** ?  ❑_1_ Yes  ❑_2_ No | | | | | | |
| --- | --- | --- | --- | --- | --- | --- |
| 1. If yes, please tell me which of the following **prescription medications** you have taken, the dose and the number of doses per day**.** | | | | | | |
| **General**  **Purpose** | **Sub-Type** | **Generic Drug Class** | **Brand Names** | **Dose(mg)** | **# of**  **Pills /**  **Dose** | **# of**  **Doses /**  **Day** |
| **Depression** |  | Amitriptyline | Elavil |  |  |  |
|  |  | Bupropion | Wellbutrin, Zyban |  |  |  |
|  |  | Citalopram | Celexa |  |  |  |
|  |  | Clomipramine | Anafranil |  |  |  |
|  |  | Desipramine | Norpramin, |  |  |  |
|  |  | Doxepin | Adapin, Sinequan |  |  |  |
|  |  | Duloxetine | Cymbalta, |  |  |  |
|  |  | Escitalopram | Cipralex, |  |  |  |
|  |  | Fluvoxamine | Luvox |  |  |  |
|  |  | Fluoxetine | Prozac |  |  |  |
|  |  | Imipramine | Tofranil |  |  |  |
|  |  | Mirtazapine | Remeron |  |  |  |
|  |  | Moclobemide | Aurorix, Manerix |  |  |  |
|  |  | Nortriptyline | Aventyl, |  |  |  |
|  |  | Paroxetine | Paxil |  |  |  |
|  |  | Phenelzine | Nardil |  |  |  |
|  |  | Sertraline | Zoloft |  |  |  |
|  |  | Tranylcypromine | Parnate |  |  |  |
|  |  | Trazodone | Desyrel |  |  |  |
|  |  | Trimipramine | Surmontil, Trimip, Tripramine |  |  |  |
|  |  | Venlafaxine | Effexor |  |  |  |
|  |  | Levomilnacipran |  |  |  |  |
|  |  | Vilazodone |  |  |  |  |
|  |  | Vortioxetine | Trintellix |  |  |  |

| **HSS-10. Other medications. Please include any eye drops or creams. Do not include non-prescription drugs.** | | | | | | |
| --- | --- | --- | --- | --- | --- | --- |
|  |  | |  | |  | |
|  |  | |  | |  | |
|  |  | |  | |  | |
|  |  | |  | |  | |
|  |  | |  | |  | |
|  |  | |  | |  | |
|  |  | |  | |  | |
|  |  | |  | |  | |
|  |  | |  | |  | |
|  |  | |  | |  | |
|  |  | |  | |  | |
|  |  | |  | |  | |
| HSS-11. a) Have you rented or purchased any supplies, aids or devices in the **past 6 months** (i.e., test strips for diabetes, glucometer, LifeLine, aids for balance like bath chairs or toilet rails, eyeglasses, hearing aids, dentures etc.)?  ❑_1_ Yes  ❑_2_ No | | | | | |  |
| 1. What have you used (include cost information – i.e., purchased, rented, rent-to-own)? | | | | | |  |
| **Item description** | | **How obtained** | | **Cost to nearest $ (monthly)** | |  |
|  | |  | |  | |  |
|  | |  | |  | |  |
|  | |  | |  | |  |
|  | |  | |  | |  |

**Patient-Provider Communication**

Thinking about any health and social services you have used in the last 6 months (e.g., family physician, nurses, social workers, specialists, hospital services, etc.), the next few questions will ask you about your experiences communicating with these providers.

In the **past 6 months**:

PPC-1. How often did health and social service providers explain things in a way that was easy to understand?

❑_1_ Never

❑_2_ Sometimes

❑_3_ Usually

❑_4_ Always

PPC-2. How often did health and social service providers show respect for what you had to say?

❑_1_ Never

❑_2_ Sometimes

❑_3_ Usually

❑_4_ Always

PPC-3. How often did providers spend enough time with you?

❑_1_ Never

❑_2_ Sometimes

❑_3_ Usually

❑_4_ Always

PPC-4. How often did providers listen carefully to you?

❑_1_ Never

❑_2_ Sometimes

❑_3_ Usually

❑_4_ Always

**Patient-Reported Experience**

CCQ-10 to what extent do you agree or disagree with the following statement: “I am given enough opportunity to help decide on care I receive”:

❑_1_ Yes, definitely

❑_2_ Yes, to some extent

❑_3_ No

❑_4_ Don’t know/not sure

IC-1 Do you know who to contact if you need to ask questions about your condition(s) or treatment(s)?

❑_1_ Yes, definitely

❑_2_ Yes, to some extent

❑_3_ No

❑_4_ Don’t know/can’t remember

IC-2 If you have questions, when can you contact the people treating and caring for you?

❑_1_ During normal working hours

❑_2_ During the evening

❑_3_ During the night

❑_4_ Weekends

❑_5_ Don’t know/not sure

IC-3 Do you feel the people treating or caring for you understand about you and your condition?

❑_1_ Yes, definitely

❑_2_ Yes, to some extent

❑_3_ No

IC-4 To what extent do you agree or disagree with the following statement…”In the last 6 months, health and social care staff have given me information about other services that are available to someone in my circumstances, including support organizations”.

❑_1_ Strongly agree

❑_2_ Agree

❑_3_ Neither agree nor disagree

❑_4_ Disagree

❑_5_ Strongly disagree

IC-5 Were you involved as much as you wanted to be in decisions about your care and support?

❑_1_ Yes, definitely

❑_2_ Yes, to some extent

❑_3_ No

IC-6 To what extent do you agree or disagree with the following statement…”I can see the impact of my involvement in on how my care is delivered”.

❑_1_ Strongly agree

❑_2_ Agree

❑_3_ Neither agree nor disagree

❑_4_ Disagree

❑_5_ Strongly disagree

IC-7 Do health and social care services help you to live the life you want as far as possible?

❑_1_ Yes, definitely

❑_2_ Yes, to some extent

❑_3_ No

IC-8 When health or social care staff plan care or treatment for you, does it happen?

❑_1_ Yes, it happens all of the time

❑_2_ It happens most of the time

❑_3_ It happens some of the time

❑_4_ No

IC-9 To what extent do you agree or disagree with the following statement…“My care and support is reviewed as often as it should be”.

❑_1_ Strongly agree

❑_2_ Agree

❑_3_ Neither agree nor disagree

❑_4_ Disagree

❑_5_ Strongly disagree

IC-10 To what extent do you agree or disagree with the following statement…“My medicines are thoroughly reviewed as often as they should be”.

❑_1_ Strongly agree

❑_2_ Agree

❑_3_ Neither agree nor disagree

❑_4_ Disagree

❑_5_ Strongly disagree

IC-11 Do you have a named health or social care professional who coordinates your care and support?

❑_1_ Yes

❑_2_ No, I coordinate my own care and support

❑_3_ Don’t know/not sure

IC-12 Do all the different people treating and caring for you work well together to give you the best possible care and support?

❑_1_ Yes, all of them work well together

❑_2_ Most of them work well together

❑_3_ Some of them work well together

❑_4_ No, they do not work well together

❑_5_ Don’t know/not sure

IC-13 Have all your needs been assessed?

❑_1_ All of my needs have been assessed

❑_2_ Some of my needs have been assessed

❑_3_ None of my needs have been assessed

❑_4_ Don’t know/can’t remember

IC-14 Were your family or caregiver involved in decisions about your care and support as much as you wanted them to be?

❑_1_ Yes, definitely

❑_2_ Yes, to some extent

❑_3_ No

❑_4_ There were no family or caregivers available to be involved

❑_5_ I didn’t want my family or caregiver to be involved in decisions about my care and support

IC-15 Overall, do you feel that your caregiver/family has had as much support from health and social services as they needed?

❑_1_ Yes, they have had as much support as they needed

❑_2_ They have had some support but not as much as they needed

❑_3_ No, they have had little or no support

❑_4_ They did not want/need support

❑_5_ There are no family members or caregivers to support
