# Supplementary material for: Study protocol for a hospital-to-home transitional care intervention for older adults with multiple chronic conditions and depressive symptoms: a pragmatic effectiveness-implementation trial
Source: BMC Geriatr. 2020 Jul 10;20:240. doi: 10.1186/s12877-020-01638-0 (PMC7350576; doi:10.1186/s12877-020-01638-0)
Supplement: Supplementary file 2 — Additional file 2. Questionnaire to measure the effectiveness of the CAST intervention on caregivers. This file includes the questionnaire that was used to measure the effectiveness of the CAST intervention on caregiver outcomes from baseline to 60 and 12-months. [file 12877_2020_1638_MOESM2_ESM.docx]

**Additional File 2**

**Questionnaire to measure the effectiveness of the CAST intervention on caregivers.**

**Sociodemographic Questionnaire**

1. How do you describe your gender?

❑_1_ Male

❑_2_ Female

❑_3_ Other (please specify): __________________________

❑_4_ Prefer not to answer

2. What is your date of birth (dd/mmm/yyyy)? ______/_______ /________

3. What is your current relationship status?

❑_1_ Never married

❑_2_ Married/living with a partner

❑_3_ Widowed

❑_4_ Separated

❑_5_ Divorced/annulled

4. What is the highest level of education that you have completed?

❑_1_ No schooling

❑_2_ Elementary school (8^th^ grade/less)

❑_3_ Did not complete secondary or high school

❑_4_ Completed secondary school or high school

❑_5_ Had some university/college education

❑_6_ Completed a community college, technical college, or post-secondary program (e.g. trade, technical or vocational school, CEGEP)

❑_7_ Completed a bachelor’s degree (e.g. B.A., B.Sc., B.S.N.)

❑_8_ Completed a graduate degree or professional degree (e.g. MD, DDS, DMD, DVM, OD, Masters, PhD)

5. Please provide your **current** employment status by choosing the main option that applies to you.

❑_1_ Employed full-time (including self-employed or on a work training program; 30 or more hours each week)

❑_2_ Employed part-time (including self-employed or on a work training program; under 30 hours each week)

❑_3_ Unemployed and looking for work

❑_4_ At school or in full-time education

❑_5_ Unable to work due to a long-term sickness or disability

❑_6_ Looking after your home/family

❑_7_ Retired from paid work

❑_8_ Doing something else

| 6. Please estimate in which of the following groups your total annual household income falls? | | |
| --- | --- | --- |
| ❑_1_ Less than $5,000 | ❑_6_ $30,000 to less than $40,000 | ❑_11_ $80,000 to less than $90,000 |
| ❑_2_ $5,000 to less than $10,000 | ❑_7_ $40,000 to less than $50,000 | ❑_12_ $90,000 to less than $100,000 |
| ❑_3_ $10,000 to less than $15,000 | ❑_8_ $50,000 to less than $60,000 | ❑_13_ $100,000 to less than $150,000 |
| ❑_4_ $15,000 to less than $20,000 | ❑_9_ $60,000 to less than $70,000 | ❑_14_ $150,000 and over |
| ❑_5_ $20,000 to less than $30,000 | ❑_10_ $70,000 to less than $80,000 | ❑_15_ Prefer not to answer |

7. Are you

❑_1_ Canadian Citizen

❑_2_ Indigenous (First Nations, Inuit, or Métis)

❑_3_ Landed immigrant

❑_4_ Other (please specify): ___________________________

8. a) What is your ethnic background? (select all that apply)

❑_1_ African

❑_2_ Asian (Chinese, Japanese, Korean, etc.)

❑_3_ Caribbean

❑_4_ European

❑_5_ Latin, Central or South American

❑_6_ Indigenous (First Nations, Inuit, or Métis)

❑_7_ South Asian (East Indian, Pakistani, Sri-Lankan, etc.)

❑_8_ Southeast Asian (Filipino, Cambodian, Laotian, Malaysian, Vietnamese, etc.)

❑_9_ West Asian or Middle Eastern (Afghan, Iranian, etc.)

❑_10_ Other (please specify): ___________________________

b) If Indigenous:

❑_1_ First Nations

❑_2_ Métis

❑_3_ Inuk

❑_4_ Other (please specify): ___________________________

9. What language(s) do you usually speak at home? (Select as all that apply)

❑_1_ English

❑_2_ French

❑_3_ Other language (please specify): _________________________

**You are a family member or friend of a study participant who was recently discharged home from the hospital. Please tell us more about your relationship to the participant and the support you provide.**

1. What is your relationship to the participant? I am a...

❑_1_ Spouse/partner

❑_2_ Brother or sister

❑_3_ Son or daughter

❑_4_ Son-in-law or daughter-in-law

❑_5_ Grandson or granddaughter

❑_6_ Friend

❑_7_ Neighbor

❑_8_ Other (please specify): ______________________________________

2. Do you live with the participant?

❑_1_ Yes

❑_2_ No

3. What kind of support do you provide for the participant? (Check all that apply)

❑_1_ Advice or emotional support

❑_2_ Assistance with tasks such as laundry, shopping, cooking, house cleaning, banking,

taking medications, transportation, etc.

❑_3_ Assistance with personal care such as bathing, dressing, grooming, toileting, feeding, assistance with moving around, etc.

❑_4_ Other (please specify): _____________________________________________________ ______________________________________________________________________________

4. How and/or why did you become a caregiver or support person to the participant?

______________________________________________________________________________

______________________________________________________________________________

______________________________________________________________________________

______________________________________________________________________________

5. Would you consider that your support/caregiving responsibilities are:

❑_1_ Occasional

❑_2_ Part-time

❑_3_ Full-time

6. Do you receive any kind of support (i.e. caregiver relief, respite, informal) for yourself for your caregiving activities/responsibilities?

❑_1_ Yes

❑_2_ No

**If yes**, please describe the type of support you receive:

a) Informal (i.e. family, friends):

______________________________________________________________________________

______________________________________________________________________________

b) Formal (i.e. paid service providers, support program or group):

______________________________________________________________________________

______________________________________________________________________________

**The next questions are about your social relationships and activities. Please select which answer is closest to your situation.**

1. There is someone I feel close to who makes me feel secure.

❑_1_ Strongly disagree

❑_2_ Disagree

❑_3_ Somewhat disagree

❑_4_ Neutral

❑_5_ Somewhat agree

❑_6_ Agree

❑_7_ Strongly agree

2. I belong to a group in which I feel important.

❑_1_ Strongly disagree

❑_2_ Disagree

❑_3_ Somewhat disagree

❑_4_ Neutral

❑_5_ Somewhat agree

❑_6_ Agree

❑_7_ Strongly agree

3. People let me know what I do well at my daily responsibilities (job, homemaking).

❑_1_ Strongly disagree

❑_2_ Disagree

❑_3_ Somewhat disagree

❑_4_ Neutral

❑_5_ Somewhat agree

❑_6_ Agree

❑_7_ Strongly agree

4. I have enough contact with the person who makes me feel special.

❑_1_ Strongly disagree

❑_2_ Disagree

❑_3_ Somewhat disagree

❑_4_ Neutral

❑_5_ Somewhat agree

❑_6_ Agree

❑_7_ Strongly agree

5. I spend time with others who have the same interests that I do.

❑_1_ Strongly disagree

❑_2_ Disagree

❑_3_ Somewhat disagree

❑_4_ Neutral

❑_5_ Somewhat agree

❑_6_ Agree

❑_7_ Strongly agree

6. Others let me know that they enjoy being with me (projects).

❑_1_ Strongly disagree

❑_2_ Disagree

❑_3_ Somewhat disagree

❑_4_ Neutral

❑_5_ Somewhat agree

❑_6_ Agree

❑_7_ Strongly agree

7. There are people who are available if I need help over an extended period of time.

❑_1_ Strongly disagree

❑_2_ Disagree

❑_3_ Somewhat disagree

❑_4_ Neutral

❑_5_ Somewhat agree

❑_6_ Agree

❑_7_ Strongly agree

8. Among my group of friends we do favours for each other.

❑_1_ Strongly disagree

❑_2_ Disagree

❑_3_ Somewhat disagree

❑_4_ Neutral

❑_5_ Somewhat agree

❑_6_ Agree

❑_7_ Strongly agree

9. I have the opportunity to encourage others to develop their interests and skills.

❑_1_ Strongly disagree

❑_2_ Disagree

❑_3_ Somewhat disagree

❑_4_ Neutral

❑_5_ Somewhat agree

❑_6_ Agree

❑_7_ Strongly agree

10. I have relatives or friends that will help me out even if I can’t pay them back.

❑_1_ Strongly disagree

❑_2_ Disagree

❑_3_ Somewhat disagree

❑_4_ Neutral

❑_5_ Somewhat agree

❑_6_ Agree

❑_7_ Strongly agree

11. When I am upset, there is someone I can be with who lets me be myself.

❑_1_ Strongly disagree

❑_2_ Disagree

❑_3_ Somewhat disagree

❑_4_ Neutral

❑_5_ Somewhat agree

❑_6_ Agree

❑_7_ Strongly agree

12. I know that others appreciate me as a person.

❑_1_ Strongly disagree

❑_2_ Disagree

❑_3_ Somewhat disagree

❑_4_ Neutral

❑_5_ Somewhat agree

❑_6_ Agree

❑_7_ Strongly agree

13. There is someone who loves and cares about me.

❑_1_ Strongly disagree

❑_2_ Disagree

❑_3_ Somewhat disagree

❑_4_ Neutral

❑_5_ Somewhat agree

❑_6_ Agree

❑_7_ Strongly agree

14. I have people to share social events and fun activities with.

❑_1_ Strongly disagree

❑_2_ Disagree

❑_3_ Somewhat disagree

❑_4_ Neutral

❑_5_ Somewhat agree

❑_6_ Agree

❑_7_ Strongly agree

15. I have a sense of being needed by another person.

❑_1_ Strongly disagree

❑_2_ Disagree

❑_3_ Somewhat disagree

❑_4_ Neutral

❑_5_ Somewhat agree

❑_6_ Agree

❑_7_ Strongly agree

**Here is a list of things that other caregivers have found to be difficult. Please check the box that applies to you. We have included some examples that are common caregiver experiences to help you think about each item. Your situation may be slightly different, but the item could still apply.**

|  | **Yes, on a regular basis** | **Yes, sometimes** | **No** |
| --- | --- | --- | --- |
| 1. My sleep is disturbed. *(For example: the person I care for is in and out of bed or wanders around at night.)* | ❑_2_ | ❑_1_ | ❑_0_ |
| 2. Caregiving is inconvenient. *(For example: helping takes so much time or it’s a long drive over to help.)* | ❑_2_ | ❑_1_ | ❑_0_ |
| 3. Caregiving is a physical strain.  *(For example: lifting in and out of a chair; effort or concentration is required.)* | ❑_2_ | ❑_1_ | ❑_0_ |
| 4. Caregiving is confining. *(For example: helping restricts free time or I cannot go visiting.)* | ❑_2_ | ❑_1_ | ❑_0_ |
| 5. There have been family adjustments. *(For example: helping has disrupted my routine; there has been no privacy.)* | ❑_2_ | ❑_1_ | ❑_0_ |
| 6. There have been changes in personal plans.  *(For example: I had to turn down a job; I could not go on vacation.)* | ❑_2_ | ❑_1_ | ❑_0_ |
| 7. There have been other demands on my time. *(For example: other family members need me.)* | ❑_2_ | ❑_1_ | ❑_0_ |
| 8. There have been emotional adjustments. *(For example: severe arguments about caregiving.)* | ❑_2_ | ❑_1_ | ❑_0_ |
| 9. Some behaviour is upsetting. *(For example: incontinence; the person cared for has trouble remembering things; or the person I care for accuses people of taking things.)* | ❑_2_ | ❑_1_ | ❑_0_ |
| 10. It is upsetting to find the person I care for has changed so much from his/her former self.  *(For example, he/she is a different person than he/she used to be.)* | ❑_2_ | ❑_1_ | ❑_0_ |
| 11. There have been work adjustments. *(For example: I have to take time off for caregiving duties.)* | ❑_2_ | ❑_1_ | ❑_0_ |
| 12. Caregiving is a financial strain. | ❑_2_ | ❑_1_ | ❑_0_ |
| 13. I feel completely overwhelmed. *(For example: I worry about the person I care for; I have concerns about how I will manage.)* | ❑_2_ | ❑_1_ | ❑_0_ |

**The following questions ask for your views about your health—how you feel and how well you are able to do your usual activities. There are no right or wrong answers; please choose the answer that best fits your life right now.**

1. In general, would you say your health is:

❑_1_ Excellent

❑_2_ Very good

❑_3_ Good

❑_4_ Fair

❑_5_ Poor

2. The following questions are about activities you might do during a typical day. Does your health limit you in these activities? If so, how much?

a) **Moderate activities**, such as moving a table, pushing a vacuum cleaner, bowling or playing golf?

❑_1_ Yes, limited a lot

❑_2_ Yes, limited a little

❑_3_ No, not limited at all

b) Climbing **several** flights of stairs?

❑_1_ Yes, limited a lot

❑_2_ Yes, limited a little

❑_3_ No, not limited at all

3. During the past four (4) weeks, have you had any of the following problems with your work or other regular daily activities as a result of your physical health?

a) Accomplished **less** than you would like.

❑_1_ Yes, all of the time

❑_2_ Yes, most of the time

❑_3_ Yes, some of the time

❑_4_ Yes, a little of the time

❑_5_ No, none of the time

b) Were limited in the **kind** of work or other activities.

❑_1_ Yes, all of the time

❑_2_ Yes, most of the time

❑_3_ Yes, some of the time

❑_4_ Yes, a little of the time

❑_5_ No, none of the time

4. During the past four (4) weeks, have you had any of the following problems with your work or other regular daily activities as a result of any emotional problems (such as feeling depressed or anxious)?

a) Accomplished **less** than you would like.

❑_1_ Yes, all of the time

❑_2_ Yes, most of the time

❑_3_ Yes, some of the time

❑_4_ Yes, a little of the time

❑_5_ No, none of the time

b) Didn’t do work or other activities as **carefully** as usual.

❑_1_ Yes, all of the time

❑_2_ Yes, most of the time

❑_3_ Yes, some of the time

❑_4_ Yes, a little of the time

❑_5_ No, none of the time

5. During the past four (4) weeks, how much did pain interfere with your normal work (including both work outside the home and housework)?

❑_1_ Not at all

❑_2_ A little bit

❑_3_ Moderately

❑_4_ Quite a bit

❑_5_ Extremely

6. These questions are about how you feel and how things have been with you during the past four (4) weeks. For each question, please give the one answer that comes closest to the way you have been feeling.

How much of the time during the past four (4) weeks:

a) Have you felt calm and peaceful?

❑_1_ All of the time

❑_2_ Most of the time

❑_3_ A good bit of the time

❑_4_ Some of the time

❑_5_ A little of the time

❑_6_ None of the time

b) Did you have a lot of energy?

❑_1_ All of the time

❑_2_ Most of the time

❑_3_ A good bit of the time

❑_4_ Some of the time

❑_5_ A little of the time

❑_6_ None of the time

c) Have you felt downhearted and blue?

❑_1_ All of the time

❑_2_ Most of the time

❑_3_ A good bit of the time

❑_4_ Some of the time

❑_5_ A little of the time

❑_6_ None of the time

During the past four (4) weeks:

7. How much of the time has your physical health or emotional problems interfered with your social activities (like visiting with friends, relatives, etc.)?

❑_1_ All of the time

❑_2_ Most of the time

❑_3_ Some of the time

❑_4_ A little of the time

❑_5_ None of the time

Now, we’d like to ask you some questions about how your health may have changed.

8. Compared to one year ago, how would you rate your physical health in general now?

❑_1_ Much better

❑_2_ Slightly better

❑_3_ About the same

❑_4_ Slightly worse

❑_5_ Much worse

9. Compared to one year ago, how would you rate your emotional problems (such as feeling anxious, depressed or irritable) now?

❑_1_ Much better

❑_2_ Slightly better

❑_3_ About the same

❑_4_ Slightly worse

❑_5_ Much worse

**Now we would like you to tell us about any other health conditions you may have.**

a) Do you have any of the following types of health problems?

| **Chronic condition** | **Yes** | **No** | **b) If yes, for 6 months or longer?** |
| --- | --- | --- | --- |
| Hypertension (high blood pressure) | ❑_1_ | ❑_2_ | ❑_1_ Yes  ❑_2_ No |
| Stomach problem (i.e., reflux or peptic ulcer symptoms) | ❑_1_ | ❑_2_ | ❑_1_ Yes  ❑_2_ No |
| Depression | ❑_1_ | ❑_2_ | ❑_1_ Yes  ❑_2_ No |
| Thyroid disorder | ❑_1_ | ❑_2_ | ❑_1_ Yes  ❑_2_ No |
| Chronic musculoskeletal condition causing pain or limitation | ❑_1_ | ❑_2_ | ❑_1_ Yes  ❑_2_ No |
| Osteoarthritis or other arthritis | ❑_1_ | ❑_2_ | ❑_1_ Yes  ❑_2_ No |
| Osteoporosis | ❑_1_ | ❑_2_ | ❑_1_ Yes  ❑_2_ No |
| Asthma or lung problem, like chronic bronchitis, emphysema, or COPD (chronic obstructive pulmonary disorder) | ❑_1_ | ❑_2_ | ❑_1_ Yes  ❑_2_ No |
| Cancer in the past 5 years (including melanoma, but not other skin cancers) | ❑_1_ | ❑_2_ | ❑_1_ Yes  ❑_2_ No |
| Heart failure (including heart valve disease or replacement) | ❑_1_ | ❑_2_ | ❑_1_ Yes  ❑_2_ No |
| Hyperlipidemia (high cholesterol) | ❑_1_ | ❑_2_ | ❑_1_ Yes  ❑_2_ No |
| Obesity | ❑_1_ | ❑_2_ | ❑_1_ Yes  ❑_2_ No |
| Colon problem (irritable bowel, Crohn’s disease, ulcerative colitis, diverticulosis) | ❑_1_ | ❑_2_ | ❑_1_ Yes  ❑_2_ No |
| Anxiety | ❑_1_ | ❑_2_ | ❑_1_ Yes  ❑_2_ No |
| Diabetes | ❑_1_ | ❑_2_ | ❑_1_ Yes  ❑_2_ No |
| Chronic liver disease (including chronic hepatitis or cirrhosis) | ❑_1_ | ❑_2_ | ❑_1_ Yes  ❑_2_ No |
| Chronic urinary problem | ❑_1_ | ❑_2_ | ❑_1_ Yes  ❑_2_ No |
| Chronic kidney disease or failure | ❑_1_ | ❑_2_ | ❑_1_ Yes  ❑_2_ No |
| Cardiovascular disease (including angina, previous heart attack, atrial fibrillation, lower limbs circulation problems) | ❑_1_ | ❑_2_ | ❑_1_ Yes  ❑_2_ No |
| Alzheimer disease or another form of dementia | ❑_1_ | ❑_2_ | ❑_1_ Yes  ❑_2_ No |
| Stroke (cerebrovascular accident or transient ischemic attack) | ❑_1_ | ❑_2_ | ❑_1_ Yes  ❑_2_ No |
| HIV | ❑_1_ | ❑_2_ | ❑_1_ Yes  ❑_2_ No |
| Other (please specify): ________________________ | ❑_1_ | ❑_2_ | ❑_1_ Yes  ❑_2_ No |
| Other (please specify): ________________________ | ❑_1_ | ❑_2_ | ❑_1_ Yes  ❑_2_ No |
| Other (please specify): ________________________ | ❑_1_ | ❑_2_ | ❑_1_ Yes  ❑_2_ No |
| Other (please specify): ________________________ | ❑_1_ | ❑_2_ | ❑_1_ Yes  ❑_2_ No |

Please list any conditions that were newly diagnosed within the **past 6 months**.
______________________________________________________________________________

______________________________________________________________________________

______________________________________________________________________________

**Below is a list of the ways you might have felt or behaved recently. For each of the following statements, please check the box which best describes how often you have felt this way during the last week.**

| **During the last week:** | Rarely or none of the time  (less than  1 day) | Some or a little of the time  (1-2 days) | Occasionally or moderate  (3-4 days) | Most or  all of the time  (5-7 days) |
| --- | --- | --- | --- | --- |
| 1. I was bothered by things that usually don’t bother me. | ❑_0_ | ❑_1_ | ❑_2_ | ❑_3_ |
| 2. I had trouble keeping my mind on what I was doing. | ❑_0_ | ❑_1_ | ❑_2_ | ❑_3_ |
| 3. I felt depressed. | ❑_0_ | ❑_1_ | ❑_2_ | ❑_3_ |
| 4. I felt that everything I did was an effort. | ❑_0_ | ❑_1_ | ❑_2_ | ❑_3_ |
| 5. I felt hopeful about the future. | ❑_0_ | ❑_1_ | ❑_2_ | ❑_3_ |
| 6. I felt fearful. | ❑_0_ | ❑_1_ | ❑_2_ | ❑_3_ |
| 7. My sleep was restless. | ❑_0_ | ❑_1_ | ❑_2_ | ❑_3_ |
| 8. I was happy. | ❑_0_ | ❑_1_ | ❑_2_ | ❑_3_ |
| 9. I felt lonely. | ❑_0_ | ❑_1_ | ❑_2_ | ❑_3_ |
| 10. I could not get *“going”*. | ❑_0_ | ❑_1_ | ❑_2_ | ❑_3_ |

| Over the **last 2 weeks**, how often have you been bothered by the following problems? | **Not at all** | **Several days** | **More than half the days** | **Nearly every day** |
| --- | --- | --- | --- | --- |
| Feeling nervous, anxious, or on edge | ❑_0_ | ❑_1_ | ❑_2_ | ❑_3_ |
| Not being able to stop or control worrying | ❑_0_ | ❑_1_ | ❑_2_ | ❑_3_ |
| Worrying too much about different things | ❑_0_ | ❑_1_ | ❑_2_ | ❑_3_ |
| Trouble relaxing | ❑_0_ | ❑_1_ | ❑_2_ | ❑_3_ |
| Being so restless that it is hard to sit still | ❑_0_ | ❑_1_ | ❑_2_ | ❑_3_ |
| Becoming easily annoyed or irritable | ❑_0_ | ❑_1_ | ❑_2_ | ❑_3_ |
| Feeling afraid as if something awful might happen | ❑_0_ | ❑_1_ | ❑_2_ | ❑_3_ |

*If you indicated that you have been bothered by any of the above problems*:

How difficult have these problems made it for you to do your work, take care of things at home, or get along with other people?

❑_1_ Not difficult at all

❑_2_ Somewhat difficult

❑_3_ Very difficult

❑_4_ Extremely difficult

**The following questions are about the health and social services you have used in the past 6 months. Please only include visits and service use related to YOUR health. Do not count visits and services related to the health of the person you support.**

1. In the **past 6 months**, have you seen your family physician (or a doctor at a walk-in clinic)?

❑_1_ Yes

❑_2_ No

**If yes**, how many times? ___________

2. In the **past 6 months**, have you seen any physician specialists?

*Specialists may include: *allergist, cardiologist, dentist, dermatologist, ear/nose/throat specialist, endocrinologist, gastroenterologist, geriatrician, nephrologist, neurologist, oncologist, ophthalmologist, optometrist, physiatrist, podiatrist, psychiatrist, radiologist, respirologist, rheumatologist, orthopedic surgeon, urologist, or any other specialists*

❑_1_ Yes

❑_2_ No

**If yes**, please complete the following table:

| Type of specialist*****  (or name and location if unknown) | Number of visits in the **past 6 months** | Did you have any out-of-pocket expenses? | If yes, how much? |
| --- | --- | --- | --- |
|  |  | ❑_1_ Yes  ❑_2_ No |  |
|  |  | ❑_1_ Yes  ❑_2_ No |  |
|  |  | ❑_1_ Yes  ❑_2_ No |  |
|  |  | ❑_1_ Yes  ❑_2_ No |  |
|  |  | ❑_1_ Yes  ❑_2_ No |  |
|  |  | ❑_1_ Yes  ❑_2_ No |  |

3. In the **past 6 months**, have you seen any other health care providers at a clinic or office?

(not including in-home visits or as a hospital patient)

*Health care providers may include: *audiologist, chiropodist, dietitian, nurse practitioner, registered nurse, foot care nurse, occupational therapist, physiotherapist, psychologist, social worker, speech-language pathologist, or any other health care professionals*

🞏_1_ Yes

🞏_2_ No

**If yes**, please complete the following table:

| Type of health care provider*  (or name and location if unknown) | Number of visits in the **past 6 months** | Did you have any out-of-pocket expenses? | If yes, how much? |
| --- | --- | --- | --- |
|  |  | ❑_1_ Yes  ❑_2_ No |  |
|  |  | ❑_1_ Yes  ❑_2_ No |  |
|  |  | ❑_1_ Yes  ❑_2_ No |  |
|  |  | ❑_1_ Yes  ❑_2_ No |  |
|  |  | ❑_1_ Yes  ❑_2_ No |  |
|  |  | ❑_1_ Yes  ❑_2_ No |  |
|  |  | ❑_1_ Yes  ❑_2_ No |  |

4. In the **past 6 months**, have you received any in-home visits by health care providers?

*Health care providers offering in-home visits may include: *care coordinator, dietitian, nurse practitioner, registered nurse, occupational therapist, personal support worker, physiotherapist, psychologist, social worker, speech-language pathologist, or any other health care providers*

🞏_1_ Yes

🞏_2_ No

**If yes**, please complete the following table:

| Type of health care provider who came to your home* | Number of visits in the **past 6 months** | Did you have any out-of-pocket expenses? | If yes, how much? |
| --- | --- | --- | --- |
|  |  | ❑_1_ Yes  ❑_2_ No |  |
|  |  | ❑_1_ Yes  ❑_2_ No |  |
|  |  | ❑_1_ Yes  ❑_2_ No |  |
|  |  | ❑_1_ Yes  ❑_2_ No |  |
|  |  | ❑_1_ Yes  ❑_2_ No |  |
|  |  | ❑_1_ Yes  ❑_2_ No |  |

5. In the **past 6 months**, have you seen any other wellness providers or participated in any other activities related to your health and well-being?

*Examples: *massage therapy, naturopathic doctor, chiropractor, meditation classes, yoga, etc.*

🞏_1_ Yes

🞏_2_ No

**If yes**, please complete the following table:

| Type of wellness provider or activity* | Number of visits in the **past 6 months** | Did you have any out-of-pocket expenses? | If yes, how much? |
| --- | --- | --- | --- |
|  |  | ❑_1_ Yes  ❑_2_ No |  |
|  |  | ❑_1_ Yes  ❑_2_ No |  |
|  |  | ❑_1_ Yes  ❑_2_ No |  |
|  |  | ❑_1_ Yes  ❑_2_ No |  |
|  |  | ❑_1_ Yes  ❑_2_ No |  |
|  |  | ❑_1_ Yes  ❑_2_ No |  |
|  |  | ❑_1_ Yes  ❑_2_ No |  |

6. In the **past 6 months**, have you received any community support services?

*Community service providers may include: *meal delivery, group dining program, house cleaning, yard work, caregiver relief, transportation services, friendly visiting, adult day program*

❑_1_ Yes

❑_2_ No

**If yes**, please complete the following table:

| Type of service* | Number of visits (times used) in the **past 6 months** | Did you have any out-of-pocket expenses? | If yes, how much? |
| --- | --- | --- | --- |
|  |  | ❑_1_ Yes  ❑_2_ No |  |
|  |  | ❑_1_ Yes  ❑_2_ No |  |
|  |  | ❑_1_ Yes  ❑_2_ No |  |
|  |  | ❑_1_ Yes  ❑_2_ No |  |

7. In the **past 6 months**, have you purchased any health care supplies, aids, or devices?

*Examples of supplies, aids, and devices include: *hearing aids, glasses, dentures, orthotics, mobility aids, raised toilet seats, grab bars, health monitoring devices (blood pressure, blood glucose), other items*

❑_1_ Yes

❑_2_  No

**If yes**, please complete the following table:

| Type of supply/aid/device purchased  in the **past 6 months*** | Approximate  total cost ($) | Did you have any out-of-pocket expenses? | If yes, how much? |
| --- | --- | --- | --- |
|  |  | ❑_1_ Yes  ❑_2_ No |  |
|  |  | ❑_1_ Yes  ❑_2_ No |  |
|  |  | ❑_1_ Yes  ❑_2_ No |  |
|  |  | ❑_1_ Yes  ❑_2_ No |  |
|  |  | ❑_1_ Yes  ❑_2_ No |  |

8. In the **past 6 months**, have you traveled to receive health care services?

❑_1_ Yes

❑_2_ No

**If yes**, number of kms: _____________

9. In the **past 6 months**, did you pay for parking while receiving health care services?

❑_1_  Yes

❑_2_ No

**If yes**, specify cost of parking:__________

10. In the **past 6 months**, have you used 911 (related to your health)?

❑_1_ Yes

❑_2_ No

**If yes**, how many times? __________

Please complete a row in the following table for each 911 call:

| Reason for 911 call | Was an ambulance used? |
| --- | --- |
|  | ❑_1_ Yes  ❑_2_ No |
|  | ❑_1_ Yes  ❑_2_  No |
|  | ❑_1_ Yes  ❑_2_ No |

11. In the **past 6 months**, have you visited the emergency room (related to your health)?

❑_1_ Yes

❑_2_ No

**If yes**, how many times? __________

Please complete a row in the following table for each emergency room visit:

| Name of hospital | Reason for emergency room visit |
| --- | --- |
|  |  |
|  |  |
|  |  |

12. In the **past 6 months**, have you been admitted to the hospital?

❑_1_ Yes

❑_2_ No

**If yes**, how many times? __________

Please complete a row in the following table for hospital admission:

| Name of hospital | Reason for admission | Number of days in hospital |
| --- | --- | --- |
|  |  |  |
|  |  |  |
|  |  |  |

13. In the **past 6 months**, have you had any lab/outpatient tests prescribed by a health professional? (Do **NOT** include tests while in the hospital or emergency room)

*Examples of lab/outpatient test include: *blood, urine, throat swab, stool test, swab/culture, ECG (heart monitoring), EEG (brain waves), breathing tests, biopsy, mammogram, scopes or other tests.*

***If you had an x-ray, ultrasound, CT scan or MRI, please tell us what body region it involved (ie, chest x-ray, CT of arm)*

❑_1_ Yes

❑_2_ No

**If yes**, please complete the following table:

| Type of lab/outpatient test performed in last **6 months** * | Number of times | Did you have any out-of-pocket expenses? | If yes, how much? |
| --- | --- | --- | --- |
|  |  | ❑_1_ Yes  ❑_2_ No |  |
|  |  | ❑_1_ Yes  ❑_2_ No |  |
|  |  | ❑_1_ Yes  ❑_2_ No |  |
|  |  | ❑_1_ No  ❑_2_ Yes |  |
|  |  | ❑_1_ No  ❑_2_ Yes |  |

14. Have you taken any prescription medications **in the past 2 days**?

❑_1_ Yes

❑_2_  No

**If yes,** please list drug name, dose (mg) per day and reason:

| Drug Name | Dose (mg) per day | Reason |
| --- | --- | --- |
|  |  |  |
|  |  |  |
|  |  |  |
|  |  |  |
|  |  |  |
|  |  |  |
|  |  |  |
|  |  |  |
|  |  |  |
|  |  |  |
|  |  |  |
|  |  |  |

15. In the **past 6 months**, have you seen a pharmacist for a medication review? (Not just picking up a prescription or any on-line resources)

❑_1_ Yes

❑_2_ No

**If yes**, how many times? ___________

16. In the **past 6 months**, please estimate the time spent performing caregiving tasks.

| Type of caregiving task performed in **past 6 months*** | Approximate number of hours **per week** | Did you have any out-of-pocket expenses? | If yes, how much?  (e.g. fee for service per hour) |
| --- | --- | --- | --- |
| 1. household tasks |  | ❑_1_ Yes  ❑_2_ No |  |
| 1. feeding, personal hygiene & toileting |  | ❑_1_ Yes  ❑_2_ No |  |
| 1. transportation |  | ❑_1_  Yes  ❑_2_ No |  |
| 1. managing finances |  | ❑_1_  Yes  ❑_2_ No |  |
| 1. supervision |  | ❑ _1_  Yes  ❑ _2_  No |  |
| 1. shopping |  | ❑_1_ Yes  ❑ _2_ No |  |

17. In the **past 6 months**, because of caregiving, have you been absent from paid work?

❑_1_ Yes

❑_2_ No

❑_3_ Not applicable

If **yes,** how many days: ____________________

18. In the **past 6 months**, because of caregiving, have you been unable to perform other unpaid activities not related to your work? (i.e. homemaking, volunteering, etc.)

❑_1_ Yes

❑_2_ No

**If yes**, what type of activities and how many days: _______________________________

________________________________________________________________________

________________________________________________________________________

________________________________________________________________________

**Each individual has different needs as a caregiver. In the next section, we will discuss some needs that others have identified. We’d like to know if you’ve had a need for service support that you were not able to get**

| 1. Are the services you are receiving meeting your physical health needs? | ❑_1_ Yes  ❑_2_ No | **If no,** what are the reasons?  ❑_1_ Challenges with moving around  ❑_2_ Transportation difficulties  ❑_3_ Didn’t know who to call or where to go  ❑_4_ Not available in the area  ❑_5_ Not available in the time needed  ❑_6_ Wait list too long  ❑_7_ Didn’t bother/decided not to seek care  ❑_8_ No respite care  ❑_9_ Language barrier  ❑_10_ Financial barrier (e.g., service not covered by provincial/federal/personal health insurance)  ❑_11_ Ineligible for available services  ❑_12_ Other (specify)_______________________ |
| --- | --- | --- |
| 2. Are the services you are receiving meeting your emotional or mental health needs? | ❑_1_ Yes  ❑_2_ No | **If no,** what are the reasons?  ❑_1_ Challenges with moving around  ❑_2_ Transportation difficulties  ❑_3_ Didn’t know who to call or where to go  ❑_4_ Not available in the area  ❑_5_ Not available in the time needed  ❑_6_ Wait list too long  ❑_7_ Didn’t bother/decided not to seek care  ❑_8_ No respite care  ❑_9_ Language barrier  ❑_10_ Financial barrier (e.g., service not covered by provincial/federal/personal health insurance)  ❑_11_ Ineligible for available services  ❑_12_ Other (specify)_______________________ |
| 3. Are the services you are receiving meeting your personal care needs (e.g., bathing, dressing)? | ❑_1_ Yes  ❑_2_ No | **If no,** what are the reasons?  ❑_1_ Challenges with moving around  ❑_2_ Transportation difficulties  ❑_3_ Didn’t know who to call or where to go  ❑_4_ Not available in the area  ❑_5_ Not available in the time needed  ❑_6_ Wait list too long  ❑_7_ Didn’t bother/decided not to seek care  ❑_8_ No respite care  ❑_9_ Language barrier  ❑_10_ Financial barrier (e.g., service not covered by provincial/federal/personal health insurance)  ❑_11_ Ineligible for available services  ❑_12_ Other (specify)_______________________ |
| 4. Are the services you are receiving meeting your housekeeping needs (e.g., food preparation, laundry)? | ❑_1_ Yes  ❑_2_ No | **If no,** what are the reasons?  ❑_1_ Challenges with moving around  ❑_2_ Transportation difficulties  ❑_3_ Didn’t know who to call or where to go  ❑_4_ Not available in the area  ❑_5_ Not available in the time needed  ❑_6_ Wait list too long  ❑_7_ Didn’t bother/decided not to seek care  ❑_8_ No respite care  ❑_9_ Language barrier  ❑_10_ Financial barrier (e.g., service not covered by provincial/federal/personal health insurance)  ❑_11_ Ineligible for available services  ❑_12_ Other (specify)_______________________ |
| 5. Are the services you are receiving meeting your home maintenance needs (e.g., snow shoveling, lawn care)? | ❑_1_ Yes  ❑_2_ No | **If no,** what are the reasons?  ❑_1_ Challenges with moving around  ❑_2_ Transportation difficulties  ❑_3_ Didn’t know who to call or where to go  ❑_4_ Not available in the area  ❑_5_ Not available in the time needed  ❑_6_ Wait list too long  ❑_7_ Didn’t bother/decided not to seek care  ❑_8_ No respite care  ❑_9_ Language barrier  ❑_10_ Financial barrier (e.g., service not covered by provincial/federal/personal health insurance)  ❑_11_ Ineligible for available services  ❑_12_ Other (specify)_______________________ |
| 6. Are the services you are receiving meeting your needs in terms of support with shopping for essential items (e.g., food, medicine)? | ❑_1_ Yes  ❑_2_ No | **If no,** what are the reasons?  ❑_1_ Challenges with moving around  ❑_2_ Transportation difficulties  ❑_3_ Didn’t know who to call or where to go  ❑_4_ Not available in the area  ❑_5_ Not available in the time needed  ❑_6_ Wait list too long  ❑_7_ Didn’t bother/decided not to seek care  ❑_8_ No respite care  ❑_9_ Language barrier  ❑_10_ Financial barrier (e.g., service not covered by provincial/federal/personal health insurance)  ❑_11_ Ineligible for available services  ❑_12_ Other (specify)_______________________ |
| 7. Are the services you are receiving meeting your respite needs? | ❑_1_ Yes  ❑_2_ No | **If no,** what are the reasons?  ❑_1_ Challenges with moving around  ❑_2_ Transportation difficulties  ❑_3_ Didn’t know who to call or where to go  ❑_4_ Not available in the area  ❑_5_ Not available in the time needed  ❑_6_ Wait list too long  ❑_7_ Didn’t bother/decided not to seek care  ❑_8_ No respite care  ❑_9_ Language barrier  ❑_10_ Financial barrier (e.g., service not covered by provincial/federal/personal health insurance)  ❑_11_ Ineligible for available services  ❑_12_ Other (specify)_______________________ |
| 8. Are the services you are receiving meeting your needs in terms of caregiver telephone support (e.g., hotline)? | ❑_1_ Yes  ❑_2_ No | **If no,** what are the reasons?  ❑_1_ Challenges with moving around  ❑_2_ Transportation difficulties  ❑_3_ Didn’t know who to call or where to go  ❑_4_ Not available in the area  ❑_5_ Not available in the time needed  ❑_6_ Wait list too long  ❑_7_ Didn’t bother/decided not to seek care  ❑_8_ No respite care  ❑_9_ Language barrier  ❑_10_ Financial barrier (e.g., service not covered by provincial/federal/personal health insurance)  ❑_11_ Ineligible for available services  ❑_12_ Other (specify)_______________________ |
| 9. Are the services you are receiving meeting your other caregiver support needs (e.g., education, strategies for managing/tracking appointments, communicating with health care team)? | ❑_1_ Yes  ❑_2_ No | **If no,** what are the reasons?  ❑_1_ Challenges with moving around  ❑_2_ Transportation difficulties  ❑_3_ Didn’t know who to call or where to go  ❑_4_ Not available in the area  ❑_5_ Not available in the time needed  ❑_6_ Wait list too long  ❑_7_ Didn’t bother/decided not to seek care  ❑_8_ No respite care  ❑_9_ Language barrier  ❑_10_ Financial barrier (e.g., service not covered by provincial/federal/personal health insurance)  ❑_11_ Ineligible for available services  ❑_12_ Other (specify)_______________________ |
| 10. Are there other service needs you have had that were not met? | ❑_1_ Yes  ❑_2_ No | **If yes,** please specify what type of care needs were not met: ______________________________________  __________________________________________  **W**hat are the reasons?  ❑_1_ Challenges with moving around  ❑_2_ Transportation difficulties  ❑_3_ Didn’t know who to call or where to go  ❑_4_ Not available in the area  ❑_5_ Not available in the time needed  ❑_6_ Wait list too long  ❑_7_ Didn’t bother/decided not to seek care  ❑_8_ No respite care  ❑_9_ Language barrier  ❑_10_ Financial barrier (e.g., service not covered by provincial/federal/personal health insurance)  ❑_11_ Ineligible for available services  ❑_12_ Other (specify)_______________________ |

**Thinking about any health and social services you have used in the last 6 months (e.g., family physician, nurses, social workers, specialists, hospital services, etc.), the next few questions will ask you about your experiences communicating with these providers.**

In the **past 6 months**:

1. How often did health and social service providers explain things in a way that was easy to understand?

❑_1_ Never

❑_2_ Sometimes

❑_3_ Usually

❑_4_ Always

2. How often did health and social service providers show respect for what you had to say?

❑_1_ Never

❑_2_ Sometimes

❑_3_ Usually

❑_4_ Always

3. How often did providers spend enough time with you?

❑_1_ Never

❑_2_ Sometimes

❑_3_ Usually

❑_4_ Always

4. How often did providers listen carefully to you?

❑_1_ Never

❑_2_ Sometimes

❑_3_ Usually

❑_4_ Always

**The following set of questions will ask you to think about the care you provide.**

1. Thinking about the person you care for, do they have a named health or social care professional who coordinates their care and support?

❑_1_ Yes

❑_2_ No, I coordinate their care and support

❑_3_ Don’t know/not sure

2. Do you know who to contact if you need to ask questions about the condition or treatment of the person you care for?

❑_1_ Yes, definitely

❑_2_ Yes, to some extent

❑_3_ No

❑_4_ Don’t know/can’t remember

3. If you have questions, when can you contact the people treating and caring for you?

❑_1_ During normal working hours

❑_2_ During the evening

❑_3_ During the night

❑_4_ Weekends

❑_5_ Don’t know/not sure

4. Do you feel the people providing care or treatment understand about the person you are caring for and their condition?

❑_1_ Yes, definitely

❑_2_ Yes, to some extent

❑_3_ No

5. Do health and social care services help the person you care for to live the life they want as far as possible?

❑_1_ Yes, definitely

❑_2_ Yes, to some extent

❑_3_ No

6. Were you involved as much as you wanted to be in decisions about the care and support of the person you are caring for?

❑_1_ Yes, definitely

❑_2_ Yes, to some extent

❑_3_ No

7. To what extent do you agree or disagree with the following statement…”I can see the impact of my involvement in on how care is delivered to the person I care for”.

❑_1_ Strongly agree

❑_2_ Agree

❑_3_ Neither agree nor disagree

❑_4_ Disagree

❑_5_ Strongly disagree

8. To what extent do you agree or disagree with the following statement…”In the last 6 months, health and social care staff have given me information about other services that are available to someone in my circumstances, including support organizations”.

❑_1_ Strongly agree

❑_2_ Agree

❑_3_ Neither agree nor disagree

❑_4_ Disagree

❑_5_ Strongly disagree

9. Thinking about the person you care for, to what extent do you agree or disagree with the following statement…“Their care and support is reviewed as often as it should be”.

❑_1_ Strongly agree

❑_2_ Agree

❑_3_ Neither agree nor disagree

❑_4_ Disagree

❑_5_ Strongly disagree

10. Thinking about the person you care for, to what extent do you agree or disagree with the following statement…“Their medicines are thoroughly reviewed as often as they should be”.

❑_1_ Strongly agree

❑_2_ Agree

❑_3_ Neither agree nor disagree

❑_4_ Disagree

❑_5_ Strongly disagree

11. Thinking about the person you care for, do all the different people treating and caring for them work well together to give you the best possible care and support?

❑_1_ Yes, all of them work well together

❑_2_ Most of them work well together

❑_3_ Some of them work well together

❑_4_ No, they do not work well together

❑_5_ Don’t know/not sure

12. Thinking about the person you care for, have all of their needs been assessed?

❑_1_ All of their needs have been assessed

❑_2_ Some of their needs have been assessed

❑_3_ None of their needs have been assessed

❑_4_ Don’t know/can’t remember

**We’re now going to ask you to think about care or support provided to you as a caregiver.**

13. Have all your care or support needs as a caregiver been assessed?

❑_1_ All of my needs have been assessed

❑_2_ Some of my needs have been assessed

❑_3_ None of my needs have been assessed

❑_4_ Don’t know/can’t remember

14. Do health and social care services help you to live the life you want as far as possible?

❑_1_ Yes, definitely

❑_2_ Yes, to some extent

❑_3_ No

15. Overall, as a caregiver do you feel that you have had as much support from health and social services as you needed?

❑_1_ Yes, I have had as much support as I needed

❑_2_ I have had some support but not as much as I needed

❑_3_ No, I have had little or no support

❑_4_ I did not want/need support

16. To what extent do you agree or disagree with the following statement: “I am given enough opportunity to help decide on care I receive to support me in my caregiving role.”:

❑_1_ Yes, definitely

❑_2_ Yes, to some extent

❑_3_ No

❑_4_ Don’t know/not sure
