# Supplementary material for: Study protocol for a hospital-to-home transitional care intervention for older adults with multiple chronic conditions and depressive symptoms: a pragmatic effectiveness-implementation trial
Source: BMC Geriatr. 2020 Jul 10;20:240. doi: 10.1186/s12877-020-01638-0 (PMC7350576; doi:10.1186/s12877-020-01638-0)
Supplement: Supplementary file 3 — Additional file 3. Interview guide for older adult and caregiver participants receiving the CAST intervention. This file includes the interview guide that was developed to guide the semi-structured interviews with older adult and caregiver participants receiving the CAST intervention. [file 12877_2020_1638_MOESM3_ESM.docx]

**Additional file 3**

**Interview guide for older adult and caregiver participants receiving the CAST intervention.**

**A. Older Adult Participants**

Thank-you for agreeing to take part in an interview today. Since your release from hospital, [name], a registered nurse, has spoken with you by phone and has visited you in your home. The purpose of her phone calls and visits has been to support you on your journey from hospital to home.

I would like to ask you some questions about the phone calls and visits that you have received from [name of nurse]. Your answers will help us to learn how to better support people returning home from hospital. Your answers will be kept confidential and will not be shared with [name of nurse]. You do not have to answer all my questions if you do not want to.

**Part A: Unmet Needs**

The first set of questions that I will ask you are about **your needs.** These needs could be physical, emotional, mental (or psychological), day-to-day basic needs (e.g., food, transportation, financial, housekeeping, personal care) or other needs.

1. What did you need most when you **first** returned home from hospital?
2. How, if at all, did [name of nurse] help to fill these needs?
   1. If need filled by CAST nurse, ask: What did her help mean to you? How did her help make you feel?
   2. If need filled by participant and/or another health professional, go to question 3.
   3. If need was not filled by anyone, ask: Is this still an important need of yours?
3. What, if any, (other) needs of yours **have not been met** since returning home from hospital? Again, these needs could be physical, emotional, mental (or psychological), day-to-day basic needs (e.g., food, transportation, financial, housekeeping, personal care) or other needs.

**Part B: Home Visits and Phone Calls Provided by [name of nurse]**

Thank-you for your help so far. The next set of questions is about the **phone calls** and **home visits** from [name of nurse].

1. When [name of nurse] **visited you at your home**, what types of things did she do during those visits?
   1. What did this mean to you?
   2. How did it make you feel?
   3. What, if anything, did you find helpful about [name of nurse] visits to your home?
   4. What, if any, of your needs were met through home visits from [name of nurse]?
   5. What, if anything, was not helpful, or that you did not like, about the home visits?
2. When [name of nurse] **called you on the phone**, what types of things did you talk about?
   1. What did this mean to you?
   2. How did it make you feel?
   3. What, if anything, did you find helpful about the phone calls from [name of nurse]?
   4. What, if any, of your needs were met through phone calls from [name of nurse]?
   5. What, if anything, was not helpful, or that you did not like, about the phone calls?
3. For other people who are returning home from hospital, would you recommend that a nurse, such as [name of nurse], visit the person at home, make phone calls or both? Can you tell me why you would recommend this?

**Part C: Patient-Reported Experience and Health System Navigation**

Thank-you for your answers so far. The next set of questions is about your experience with the **care** that you received from [name of nurse]. Some of these care questions may or may not be relevant to your care. If the question is not relevant, please let me know and we will go on to the next one.

1. How, if at all, did [name of nurse]:
   1. involve you in decisions about your care?
   2. take your preferences (i.e., the way you like things done) into consideration?
   3. respect your values and beliefs?
   4. take steps to improve your mental and emotional well-being?
   5. take steps to improve your physical health?
   6. teach you how to better manage your health?
   7. help you to work through a problem?
   8. discuss your medications with you?
2. To what extent did the nurse help to address your needs or the issues that were most important to you?
3. How happy are you with the **overall care** that you received from [name of nurse]? Probe: Why do you say this?
4. Is there anything else about your experiences with [name of nurse] that you would like to add that we have not already discussed?

**Part D: Patient-Provider Communication (only if time)**

Thank-you for your help so far. The last set of questions that I will ask you are about **communication.**

1. When [name of nurse] was **speaking** to you:
   1. How easy was it for you to **hear** what she was saying?
   2. How easy was it for you to **understand** what she was saying?

If issues identified, probe: Can you tell me more?

1. How well did [name of nurse]:
   1. **listen** to you?
   2. **understand** you?
   3. **answer** your questions?
   4. **provide** information that was helpful to you?

Probe, if needed: Can you tell me more?

1. Was the information that you were given by [name of nurse] and other health professionals about care:
   1. consistent (across individuals)? How did this make you feel?
   2. confusing? How did this make you feel?
2. Were you provided any **written** information from [name of nurse]?
   1. If yes,
      1. how easy was this information to **read**?
      2. How easy was this information to **understand**?
      3. Was this written information **helpful** to you? If yes, how so?
   2. If no:
      1. Would you have liked to have received **written** information from [name of nurse]?
         1. If yes, what kind(s) of written information?
         2. If no, go to next question.
3. Is there anything about your communications with [name of nurse] that you would like to add that we have not already discussed?

Thank-you again for taking part in this interview. Your input is very helpful.

**B Caregiver Participants**

Thank-you for agreeing to take part in an interview today. Since your family member’s/friend’s release from hospital, [name], a registered nurse, has spoken with you by phone and/or has made visits to the home. The purpose of her phone calls and visits is to support people after they have been released from hospital and their families.

I would like to ask you some questions about the phone calls and home visits made by [name of nurse]. Your answers will help us to learn how to better support people returning home from hospital and their families. Your answers will be kept confidential and will not be shared with [name of nurse]. You do not have to answer all of my questions, if you do not want to.

Do you have any questions, or concerns?

**Part A: Unmet Needs**

The first set of questions that I will ask you are about **your needs.** These needs could be physical, emotional, mental (or psychological), day-to-day basic needs (e.g., food, transportation, financial, housekeeping, personal care) or other needs.

1. When your family member/friend **first** returned home from hospital, what were you **most** in need of?
2. How, if at all, did [name of nurse] help to fill this need?
   1. If need filled by CAST nurse, ask: What did her help mean to you? How did her help make you feel?
   2. If need filled by another health professional, go to question 3.
   3. If need was not filled by anyone, ask: Is this still an important need of yours?
3. What, if any, (other) needs of yours **have not been met** since your family member’s/friend’s release from hospital? Again, these needs could be physical, emotional, mental (or psychological), day-to-day basic needs (e.g., food, transportation, financial, housekeeping, personal care) or other needs.

**Part B: Home Visits and Phone Calls Provided by [name of nurse]**

Thank-you for your help so far. The next set of questions is about the **phone calls** and **home visits** from [name of nurse].

1. When [name of nurse] **visited you at your home,** what types of things did she do during those visits?
   1. What did this mean to you?
   2. How did it make you feel?
   3. What, if anything, did you find **helpful** about the home visits from [name of nurse]?
   4. What, if any, of your needs were met through home visits from [name of nurse]?
   5. What, if anything, was **not helpful**, or that you did not like, about the home visits?
2. When [name of nurse] **called you on the phone,** what types of things did you talk about?
   1. What did this mean to you?
   2. How did it make you feel?
   3. What, if anything, did you find helpful about the phone calls from [name of nurse]?
   4. What, if any, of your needs were met through phone calls from [name of nurse]?
   5. What, if anything, was not helpful, or that you did not like, about the phone calls?
3. For other people who are returning home from hospital, would you recommend that a nurse, such as [name of nurse], visit the person at home, make phone calls or both? Can you tell me why you recommend this?

**Part C: Caregiver-Reported Experience and Health System Navigation**

Thank-you for your answers so far. The next set of questions is about your experience with the **care** that you received from [name of nurse]. Some of these care questions may or may not be relevant to your care. If the question is not relevant, please let me know and we will go on to the next one.

1. How, if at all, did [name of nurse]:
   1. involve you in decisions about your care?
   2. take your preferences (i.e., the way you like things done) into consideration?
   3. respect your values and beliefs?
   4. take steps to improve your mental and emotional well-being?
   5. take steps to improve your physical health?
   6. ensure that the information given to you from other health professionals involved in your care were consistent (or the same)?
   7. teach you how to better manage your health?
   8. help you to work through a problem?
   9. discuss your medications with you?
2. To what extent did the nurse help to address your needs or the issues that were most important to you?
3. How happy are you with the **overall care** that you received from [name of nurse]? Probe: Why do you say this?
4. Is there anything about your experiences with [name of nurse] that you would like to add that we have not already discussed?

**Part D: Caregiver-Provider Communication**

Thank-you for your help so far. The last set of questions that I will ask you are about **communication.**

1. When [name of nurse] was **speaking** to you:
   1. How easy was it for you to **hear** what she was saying?
   2. How easy was it for you to **understand** what she was saying?

If issues identified, probe: Can you tell me more?

1. How well did [name of nurse]:
   1. **listen** to you?
   2. **understand** you?
   3. **answer** your questions?
   4. **provide** information that was helpful to you?

Probe, if needed: Can you tell me more?

1. Was the information given by [name of nurse] and other health professionals about care:
   1. consistent (across individuals)? How did this make you feel?
   2. confusing? How did this make you feel?
2. Were you provided any **written** information from [name of nurse]?
   1. If yes,
      1. how easy was this information to **read**?
      2. How easy was this information to **understand**?
      3. Was this written information **helpful** to you? If yes, how so?
   2. If no:
      1. Would you have liked to have received **written** information from [name of nurse]?
         1. If yes, what kind(s) of written information?
         2. If no, go to next questions.
3. Is there anything about your communications with [name of nurse] that you would like to add that we have not already discussed?

Thank-you again for taking part in this interview. Your input is very helpful.
