# Supplementary material for: Study protocol for a hospital-to-home transitional care intervention for older adults with multiple chronic conditions and depressive symptoms: a pragmatic effectiveness-implementation trial
Source: BMC Geriatr. 2020 Jul 10;20:240. doi: 10.1186/s12877-020-01638-0 (PMC7350576; doi:10.1186/s12877-020-01638-0)
Supplement: Supplementary file 4 — Additional file 4. Interview guide for Care Transition Coordinators. This file includes the interview guide that was developed to guide the semi-structured interviews with the Care Transition Coordinators who delivered the CAST intervention. [file 12877_2020_1638_MOESM4_ESM.docx]

**Additional file 4**

**Interview Guide for Care Transition Coordinator**

Thank you for taking the time to participate in our interview. I just want to start by giving you a bit of an introduction to what we will be talking about today. Today we are going to be talking about the approaches used to plan and conduct Community Assets Supporting Transitions (CAST) study. We are interested in how the program has been adapted to meet the needs of communities and what will need to happen to continue the program after the study is complete.

When we’re talking about conducting the research, we are referring to the many different stages of CASTs work in which you may have participated or been engaged, from the shaping how the study should roll out, to recruitment strategies, and collecting data, and so on. Now that you have been implementing your role for some time, we’d like to start with revisiting some questions about the CAST program.

1. Can you describe what support you have had by the research team and the extent to which this support met your needs?
   - Can you describe a recent example?
2. How confident are you that you have been able to successfully implement the intervention?
   - What gives you that level of confidence (or lack of confidence)?
3. Has the intervention been implemented according to the implementation plan? Please tell me about both successes and challenges.
   - Based on your experiences to date: how well have you been able to ensure that your care is person- and family-centred?
   - Probes: e.g., goal-centred, looked at the whole person, tailoring, partnering
4. Based on your experiences thus far, what kinds of changes or alterations have you needed to make to the intervention to work effectively in your community?
5. Have you experienced any overlaps with existing roles and supports available in your community? Please explain. How have you navigated these overlaps? What have you done to reduce or avoid duplication and/or promote better communication and coordination?
6. Based on your experiences implementing the CAST study to date, who are influential community members that have been important to get on board with the intervention?
7. Can you describe your working relationships with your community-based colleagues when implementing the intervention?
   - With colleagues in your organization? *(**only ask if RN-CTC is hired through an external agency/organization [i.e., via secondment])*
   - With colleagues in other organizations?
   - Can you tell me a story about a time you needed to work with others to solve a problem? Or to implement this intervention?
8. What steps have you taken to encourage other community providers to commit to engaging with you to support the intervention?
   - Which individuals have you targeted?
   - How have you approached them?
   - What information have you given them?
   - How frequently and how will you communicate with them?
9. How complicated is the CAST intervention?
   - Please consider the following aspects of the CAST intervention: duration, scope, intricacy, and number of steps involved and whether the intervention reflects a clear departure from previous practices.
10. What components of the CAST intervention have been implemented most successfully to date?

Probes:

- - care coordination, system navigation and case management (direct or indirect)
  - clinical assessments and screening
  - health education and health literacy
  - behavioural change support (e.g., behavioural activation, problem-solving therapy, supporting behaviour change)
  - clinical care (e.g., management of chronic conditions and depressive symptoms, includes medication reconciliation, review and management)
  - psychosocial and practical support
  - building trusting patient-caregiver-provider relationships
  - health promotion and disease prevention
  - self-management support
  - caregiver health and support

1. What components of the CAST intervention have been most challenging to implement?

Probes:

- - care coordination, system navigation and case management (direct or indirect)
  - clinical assessments and screening
  - health education and health literacy
  - behavioural change support (e.g., behavioural activation, problem-solving therapy, supporting behaviour change)
  - clinical care (e.g., management of chronic conditions and depressive symptoms, includes medication reconciliation, review and management)
  - psychosocial and practical support
  - building trusting patient-caregiver-provider relationships
  - health promotion and disease prevention
  - self-management support
  - caregiver health and support

1. What challenges have you faced connecting with or working with participants in implementing the intervention? How have you tried to address these?
2. Can you give an example of when the program made a difference to an older adult participant?
3. Can you give an example of when the program made a difference to a caregiver?
4. Do you have any additional comments or feedback on the implementation of the CAST study and your role to date that we have not yet discussed, and you would like to add?
