# Supplementary material for: Study protocol for a hospital-to-home transitional care intervention for older adults with multiple chronic conditions and depressive symptoms: a pragmatic effectiveness-implementation trial
Source: BMC Geriatr. 2020 Jul 10;20:240. doi: 10.1186/s12877-020-01638-0 (PMC7350576; doi:10.1186/s12877-020-01638-0)
Supplement: Supplementary file 5 — Additional file 5. Interview guide for Community Advisory Boards. This file includes the interview guide that was developed to guide the semi-structured interviews with the Community Advisory Boards. [file 12877_2020_1638_MOESM5_ESM.docx]

**Additional file 5**

**Community Advisory Board Interview Guide**

Thank you for taking the time to participate in our focus group. I just want to start by giving you a bit of an introduction to what we will be talking about today. Today we are going to be talking about the approaches used to plan and conduct the Community Assets Supporting Transitions (CAST) study. We are interested in how the program has been adapted to meet the needs of your specific community and what will need to happen to continue the program after the study is complete. We will also be asking you about how patients, caregivers, and other community partners have been engaged as partners in the research team.

To remind you, when we talk about engagement, we’re talking about the ways that you have participated in the work associated with the Community Assets Supporting Transitions (CAST) study.

When we talk about research work, we are referring to the many different stages of the CAST study in which you may have participated or been engaged, from the development of the partnership and research proposal, to shaping how the study should roll out, to recruitment strategies, collecting and analyzing data, to developing key messages, and so on.

We are going to start by asking you some questions about the processes used to engage patients, caregivers, and other community partners as research partners in the CAST research team.

1. Based on your experience working with the CAST study, what has been the purpose and role of engaging patients, caregivers, and other community partners in the CAST study?
2. In your opinion, what difference has patient, caregiver, and community engagement in the CAST study made?

- Probe: How has this engagement added value to the research approaches used in CAST?

1. What are the best ways to enable patient, caregiver, and community partners’ engagement in research? What needs to be in place to support the engagement of patient, caregiver, and community partners?

- Probe: What helps you to be able to be involved in the research? What do you like best about being involved in the research?

1. What makes it difficult for patients, caregivers and community partners to become engaged in research?

1. What have been your experiences of engagement in the CAST research study to date?
   - Probe: How included in discussions have you felt? How supported have you felt by the team? How respected or valued have you felt as a partner? How involved have you felt in identifying problems and gaps, working together to develop solutions, or making decisions?
2. Can you give any examples of how the study has been changed because of patient, caregiver, and community engagement?
   - Probe: Have the researchers told you that they have changed anything because of what you have said? Have there been any changes in how the CAST program was adapted, delivered, or evaluated? Have there been any changes in the CAST study tools, documents or training/support resources based on your input?
3. What impact, if any, has there been on you personally or professionally because of being involved in the research?

Next, we’re going to ask you some questions, more broadly, about the CAST program and how it has been working in your community to date.

1. What is the current stage of the research?

Probes:

- - How do you think the program is going?
  - Why do you say that?

1. Based on your understanding, has the program been implemented according to the implementation plan?

Probes:

- - [If Yes] Can you describe this?
  - [If No] Why not?

1. Do you think that this program should continue after the research project is done?
2. What, if any, aspects of the program should stay in place after the research project is done?

Probes:

- - Which ones?

1. What kinds of further changes or adaptations, if any, do you think will need to be made to the program after the study ends?
2. What is the likelihood that the program will become a part of usual practice in your community? Why?

Probes:

- - How do you feel about the plan to sustain the program in your setting?
  - Do you have any feelings of anticipation? Stress? Enthusiasm? Why?

1. Since we last spoke, have you heard stories about the experiences of participants with the program?

Probes:

- - Can you describe a specific story?

1. Those are all the questions that we have for you today. Is there anything else that you would like to share about conducting the CAST study in your community or the processes used to engage older adults and other community members as partners in the research team?

Thank you for taking part in this interview. We really value your perspective and appreciate the ideas you have shared.
